# Multitarget Drug Design for Cardiovascular Disease: Bridging Chemistry and Medicine

**DOI:** 10.1002/cmdc.70506

**Published:** 2026-10-13

**Authors:** Gyuzel Y. Mitronova, Vladimir N. Belov, Constanze Schmidt, Stephan E. Lehnart

**Affiliations:** ^1^ Translational Neuroinflammation and Automated Microscopy TNM Fraunhofer‐Institute for Translational Medicine and Pharmacology Göttingen Germany; ^2^ Department of NanoBiophotonics Max Planck Institute for Multidisciplinary Sciences Göttingen Germany; ^3^ DZHK (German Centre for Cardiovascular Research) Göttingen Germany; ^4^ Department of Cardiology & Pulmonology Heart Research Center Göttingen University Medical Center Göttingen Göttingen Germany

**Keywords:** cardiac diseases, cardiology, drug design, ligand design, multi‐target drugs

## Abstract

This review provides an overview of recent advances in the development of multitarget drugs (MTDs) for cardiac diseases, with a particular focus on emerging molecular scaffolds and their pharmacological properties. We discuss the fundamental principles of cardiac polypharmacology and multitarget drug design, highlighting key compound classes, such as 1,4‐benzothiazepines, 1,4‐dihydropyridines (DHPs), and polyphenol derivatives. Particular emphasis is placed on the relationship between the structural features and the pharmacological activity, efficacy, and therapeutic potential of these compounds.

## Graphical abstract

Many pathophysiological pathways associated with cardiovascular diseases involve multiple drug targets. This article reviews recent advances in the development of pharmacological compounds capable of simultaneously modulating multiple mechanisms. Multitarget drugs offer a promising therapeutic approach for complex cardiovascular disorders, including atrial fibrillation, heart failure, and myocardial ischemia–reperfusion injury, by simultaneously modulating multiple disease‐relevant pathways while reducing the need for polypharmacy and the associated risk of adverse drug–drug interactions. (Heart pictogram was created using Biorender, and protein structures were adapted from the RCSB PDB under accession codes 6JJU (blue), 5GOA (green), and 8WE7 (red)).

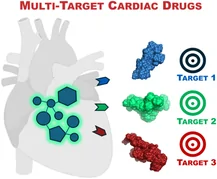

## 1 Introduction

Cardiovascular diseases (CVDs) are complex disorders characterized by multifactorial etiologies and diverse clinical manifestations. The complexity arises from the interplay of molecular, cellular, tissue, and systemic processes that converge on multiple pathophysiological pathways simultaneously [1, 2, 3, 4, 5]. Interconnected networks comprise numerous druggable targets, including receptors, ion channels, kinases, transcription factors, and enzymes, which are increasingly being explored for therapeutic interventions. Given the intricate molecular architecture underlying cardiovascular disease progression, therapeutic strategies that simultaneously modulate multiple disease‐relevant pathways have gained considerable attention. In this context, multitarget drugs (MTDs) offer several potential advantages over conventional single‐target therapies. By targeting multiple mechanisms within a single molecular entity, MTDs may enhance therapeutic efficacy, reduce the need for polypharmacy, limit the development of drug resistance, and decrease the risk of dose‐dependent adverse effects [6, 7, 8, 9].

The rationale for such approaches is further underscored by the challenges associated with conventional polypharmacy, where the concomitant administration of multiple agents may lead to unforeseen and clinically significant drug–drug interactions (DDIs). A notable example is the severe proarrhythmic interaction observed in clinical trials between the antiarrhythmic drug amiodarone and the hepatitis C antiviral sofosbuvir [10]. Mechanistic studies have recently demonstrated that this interaction can result in profound inhibition of sinoatrial node automaticity, a vitally important cardiac pacemaker tissue, through unintended blockade of the cation‐selective pore of the voltage‐gated L‐type calcium channel Ca_V_1.3 [10]. Ca^2+^ is an abundant cation and vital second messenger. Fluctuations in intracellular Ca^2+^ concentration play a crucial role in various cardiac signaling pathways. This underscores the intricate nature of pharmacological modulation within the interconnected networks of cardiac signaling.

Numerous pharmacological agents have been found to exert polypharmacological effects rather than acting with strict specificity. For example, aspirin was found to act on several molecular pathways, aside from cyclooxygenase inhibition [6, 11]. In many cases, these multiactivities were not anticipated at the time of drug discovery. However, subsequent pharmacological and biochemical studies have revealed that such unintended interactions can contribute significantly to both therapeutic efficacy and adverse side effect profiles. For example, sotagliflozin, an inhibitor of sodium‐glucose cotransporter 1 and 2 (SGLT1/SGLT2 inhibitor) [12], was found to impact p38 mitogen‐activated protein kinase (MAPK) and c‐Jun N‐terminal kinase pathways (P38MAPK and JNK), providing additional cardiac protection [13]. The structures of several established multitarget cardiac drugs including sotagliflozin [13], amiodarone [14], and verapamil [15] with indicated pharmacological targets are illustrated in Figure 1. This insight has broadened our understanding of how drugs work and highlighted the principle of multitarget interaction and the associated polypharmacology as a central concept in drug research and systems pharmacology [7, 16, 17, 18]. Over the past two decades, this insight has driven a paradigm shift from the traditional “one drug–one target” approach toward the rational design of multitarget agents capable of simultaneously modulating predefined disease‐relevant pathways [19, 20]. This transition has been particularly important for complex multifactorial disorders, such as CVDs, in which therapeutic efficacy often depends on the coordinated regulation of multiple interconnected pathophysiological mechanisms.

**FIGURE 1:**
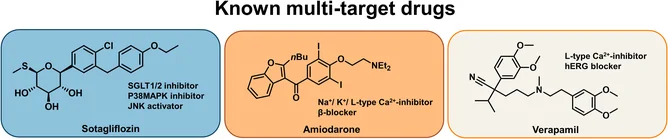
Structures of known multitarget cardiac drugs and their pharmacological targets: sotagliflozin [13], amiodarone [14] and verapamil [15].

The main challenges in developing MTDs include target selection, balancing affinity and activity, and achieving desirable pharmacokinetics. Target selection involves identifying suitable proteins or pathways that can be simultaneously modulated to achieve better therapeutic outcomes [6, 21]. To maximize the therapeutic index, ideal targets should demonstrate disease‐restricted expression or activation, permitting selective modulation of disease pathways while preserving normal cellular function and lowering the incidence of adverse events. Combining different pharmacophores can create compounds with unfavorable properties, such as poor bioavailability or the violation of drug‐likeness rules. A modification that improves the affinity for one target can unpredictably alter interactions with others, complicating optimization and requiring extensive computational and experimental modeling to maintain balanced activity [22].

In this review, we present a set of references for MTDs intended for cardiac diseases, highlighting the rational design strategies that underpin the development of these innovative therapeutics.

A literature search was conducted using renowned databases such as PubMed, Web of Science, Google Scholar, and SciFinder, applying search terms such as “MTDs, dual‐target, multitarget drug design, dual inhibitors, cardiac, cardiovascular” and various combinations thereof, with a focus on publications from the last five years. The screening process was completed in March 2026. Original research articles, reviews, and studies describing the design, synthesis, and/or biological evaluation of multitarget cardiovascular agents were included. Duplicates, non‐English articles, conference abstracts without full texts, and publications outside the review scope were excluded. Several reviews covering the potential of multitarget approaches in cardiac diseases have recently been published [8, 23–26]. Rapid progress in the field, including the approval of new drugs, novel mechanisms, updated clinical data, and innovative design paradigms, requires constant and fresh overviews, as well as new interpretations to guide future research and clinical translation of these drugs.

Several complementary strategies have been developed for the design of MTDs. In the following sections, we discuss these approaches in the context of the intricate pathophysiological networks underlying cardiovascular diseases, with particular emphasis on medicinal chemistry principles that enable the rational development of multitarget cardiovascular therapeutics.

## 2 Fragment‐Based Pharmacophore Combination or Molecular Hybridization

Molecular hybridization is an approach that integrates multiple pharmacophores into a single molecular framework to generate structurally unified hybrid compounds with potential synergistic biological activity [27, 28]. The initially adopted strategy involved creating molecules that act on several targets, which have already been independently validated as relevant for treating the same disease [6, 26, 27, 28, 29, 30, 31, 32]. Considering that the starting lead compounds have no geometric similarity or pharmacophoric elements and the targets can interact with ligands that share comparable geometry and pharmacophore patterns, we can rely on linking and fusing the frameworks of such selective molecules as a well‐known combinatorial approach to developing hybrid multitarget ligands [7, 20, 31, 32].

### 2.1 Linking Approach

In the linking approach, individual pharmacophoric fragments are selected to retain activity toward their respective targets and are subsequently connected through a linker of appropriate length, flexibility, and spatial orientation to preserve target engagement and biological activity. The key design goal is to retain the binding mode of the original fragment while adding a second activity without causing steric clashes or excessive loss of potency [33]. Linking strategy is based on the assembly of pharmacophoric units using either cleavable or non‐cleavable linkers, thereby enabling the integration of multiple biological activities within a single compound.

The following section provides an overview of the key achievements in the development of dual‐action drugs for cardiology using this approach.

*Non‐cleavable linker*. Abdelazeem et al. implemented a molecular linking strategy to design dual cyclooxygenase 2/soluble epoxide hydrolase (COX‐2/sEH) inhibitors that exhibit synergistic pharmacological activity, resulting in the concurrent attenuation of inflammatory processes and mitigation of cardiovascular risk [34]. COX‐2 and sEH are key therapeutic targets for several disorders, including cardiovascular disorders, and their inhibitors display multifaceted clinical effects [35, 36]. The combined inhibition of COX‐2 and sEH has been shown to generate synergistic therapeutic effects by suppressing inflammation while counteracting the cardiovascular risks traditionally associated with selective COX‐2 inhibition [37]. As a starting point for the design of dual inhibitors, Abdelazeem et al. selected structures similar to celecoxib, a COX‐2 inhibitor. These structures include Y‐shaped diaryl heterocycles and a sulfonamide group at the p‐position of one of the two phenyl rings (Figure 2) [34]. Importantly, the pyrazole ring has a high affinity for COX‐2. When exploring the sEH pharmacophoric moieties, the authors focused on the structures of substituted urea or amide moieties that had previously been identified as essential for sEH catalytic domain interactions [38]. In addition, trifluoromethyl phenyl, cyclohexyl, adamantyl, piperidine, and long‐chain dual amine/fatty acid substituents were selected based on their significant contribution to sEH activity [38]. In vitro studies have shown that the most potent and selective COX‐2 inhibitors, which also inhibit sEH, contain a urea bridge, suggesting that this short and rigid linker determines the conformation of the final molecules that enable binding to the targets [34]. Molecular docking revealed that the urea linker facilitated binding to the target by forming a hydrogen bond with Asp333 in the sEH catalytic site. In vitro and in vivo results showed that the compound with a *p*‐trifluoromethyl phenyl moiety directly attached to the urea linker, 4‐(1‐phenyl‐3‐(3‐(4‐(trifluoromethyl)phenyl)ureido)‐1H‐pyrazol‐5‐yl)benzenesulfonamide (PTPUP), exhibited the highest potency as a COX2/sEH dual inhibitor. It inhibited COX‐2 and sEH with IC_50_ values of 1.24 μM and 0.40 nM, respectively (Figure 2). According to the molecular docking results and comparison with the other compounds studied, this compound exhibited the highest number and variety of interactions with sEH. The authors note that hybridization between the urea group and the sulfonamide‐containing diarylpyrazole COX‐2 core represents a promising starting point for further lead optimization [34].

**FIGURE 2:**
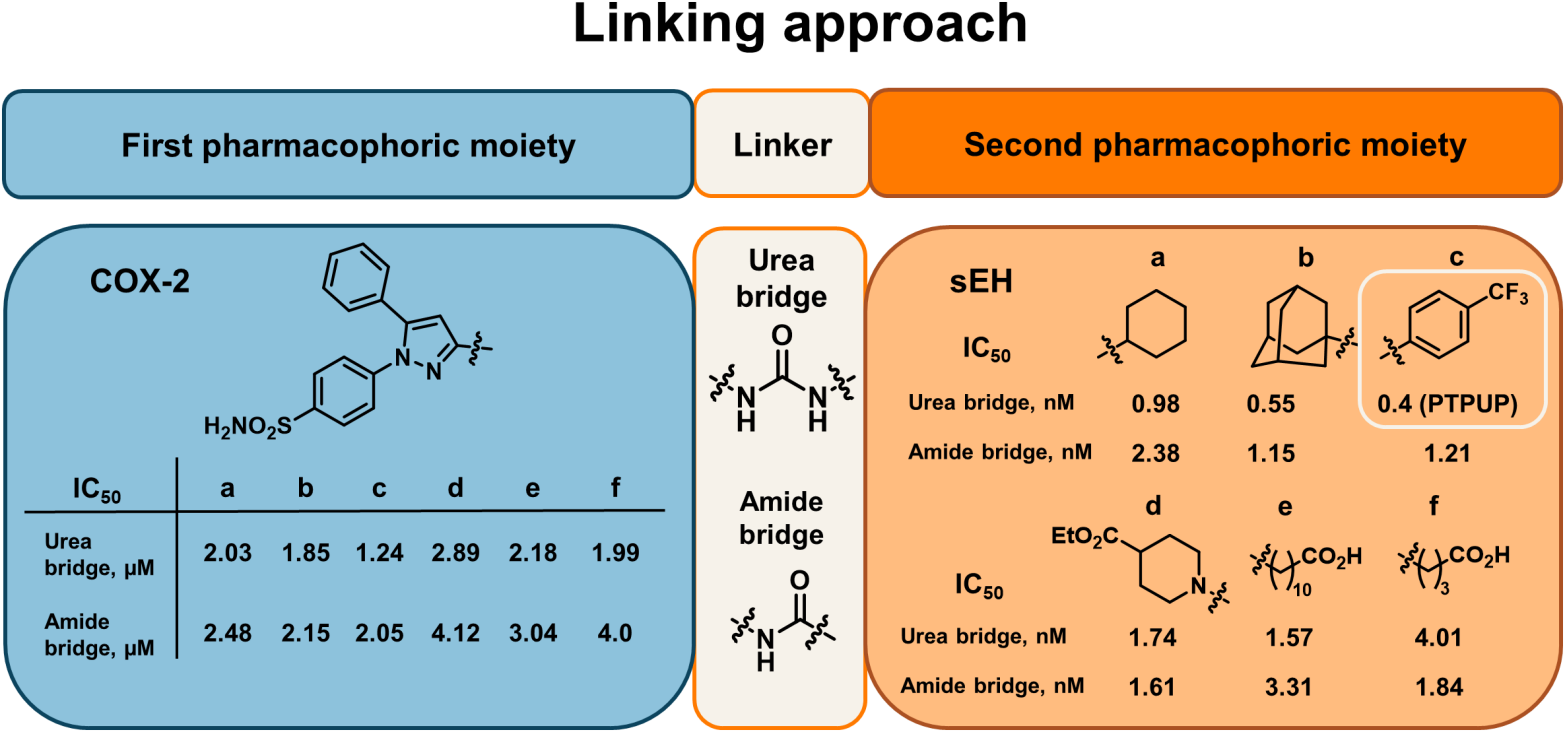
Linking approach to the development of dual COX‐2/sEH inhibitors [34]. The first pharmacophoric moiety, a COX‐2 inhibitor, is connected via a urea or amide linker to the second pharmacophoric moiety, which confers sEH inhibitory activity and consists of cyclohexyl (a), adamantyl (b), trifluoromethylphenyl (c), piperidine (d), or long‐chain fatty acid groups (e,f). The compound with a para‐trifluoromethyl phenyl moiety directly attached to the urea linker (PTPUP) was the most potent COX2/sEH dual inhibitor.

In our recent publication, we implemented a linking strategy by combining a 1,4‐benzothia‐ or 1,4‐benzoxazepine scaffold with a 3‐(3,4‐dihydroxyphenyl)‐2‐propenoic or caffeic acid (CA) residue (Figure 3) [39]. Targeting ryanodine receptor 2 (RyR2) and sarco/endoplasmic reticulum Ca^2+^‐dependent ATPase 2a (SERCA2a) is essential for the coordinated contraction and relaxation of cardiac muscles, as RyR2 mediates the rapid release of calcium (Ca^2+^) from the sarco‐endoplasmic reticulum (SER) into the cytoplasm, whereas SERCA2a transports Ca^2+^ from the cytoplasm back into the SER [39]. Pathologically increased RyR2 gain‐of‐function promotes spontaneous, uncontrolled depletion of Ca^2+^ from the SER (“leaky” channels) in resting cardiomyocytes (CMs) [40]. Reduced SERCA2a activity impairs muscle relaxation by elevating cytosolic Ca^2+^ levels above the physiological resting diastolic concentration of approximately 100 nM, leading to RyR2 channel activation at the wrong time (diastole) and pathogenic Ca^2+^ wave propagation. Therefore, the joint regulation or therapeutic modification of the net balance of these two Ca^2+^ transporter complexes is crucial for maintaining cardiac health. We used 1,4‐benzothiazepine structures, which are recognized in medical chemistry as inhibitors of dysfunctional ryanodine receptors (RyRs), to stabilize these channels and reduce abnormal Ca^2+^ leakage from the SER store [40]. These agents, including K201 (JTV519) and S‐107, restore the decreased cytosolic binding of regulatory proteins (such as FKBP12.6) to RyR2, stabilizing the closed state of the channel, an allosteric mechanism preventing pathological Ca^2+^ release [40, 41, 42, 43, 44]. Excessive Ca^2+^ loss from the SER through leaky RyR2 channels is widely recognized as a key mechanism underlying the development of atrial fibrillation (AF), the most common sustained cardiac arrhythmia, as well as an important contributor to ventricular arrhythmogenesis and the risk of potentially life‐threatening ventricular tachyarrhythmias [42, 45, 46, 47]. We selected the second pharmacophore (CA) from the low‐molecular‐weight compounds that bind directly to SERCA [48, 49]. In vitro studies showed that covalent bonding of two moieties results in compounds that can reduce the RyR2 open‐state probability (up to 6%) and accelerate SERCA2 (up to 18%) [39]. The 1,4‐benzothiazepine derivatives **12** and **13**, which contain polar N‐B moieties in their linking segments, exhibited the best dual activity against RyR2 and SERCA2a (Figure 3). The IC_50_ values for ATPase activity were in the micromolar range at 2.3 and 23.1 μM, respectively [39]. In addition, we evaluated the effects of the compounds on the voltage‐gated sodium channel Na_V_1.5 as the structural similarities between RyR2 and Na_V_1.5 suggest a possible functional overlap or shared binding characteristics for drug molecules [50]. After the addition of compounds **12** and **13**, we observed a 14% and 17% decrease in Na_V_1.5 activity, respectively. Na_V_1.5 blockade slows conduction and suppresses re‐entry circuits. In combination with RyR2 stabilization and SERCA2a activation, they simultaneously target several mechanisms underlying the development of arrhythmia. Notably, the clinical acceptability of Na_V_1.5 inhibitors depends on their efficacy, dose dependence, and selectivity. However, compounds that inhibit the peak Na^+^ current may also suppress the late Na^+^ current (I_NaL_), which arises from the slow activation or incomplete inactivation of Na_V_1.5 channels [51]. Genetic mutations or acquired Na^+^ channel dysfunction typically enhance this pathological current. Pharmacological inhibition of I_NaL_ is often more pronounced than that of the peak Na^+^ current, which is highly desirable from a therapeutic standpoint [52]. Further investigations are needed to elucidate the mechanisms by which CA derivatives modulate I_NaL_ and the resulting functional consequences. To advance these compounds toward clinical translation, further validation in complex preclinical models, such as disease‐relevant cardiac organoids and in vivo animal models, is required. Comprehensive off‐target screening (particularly against hERG and Ca_V_1.2 channels) and detailed electrophysiological characterization are necessary to confirm the favorable therapeutic index of these compounds. The inhibition of gain‐of‐function activity in the homologous skeletal muscle RyR1 channel by these multitarget compounds is theoretically anticipated but remains to be experimentally verified. Although structural homology suggests plausible RyR1 engagement, its physiological relevance must be contextualized within the skeletal muscle fiber‐type heterogeneity and the associated variations in SERCA1‐dependent SR Ca^2+^ uptake. Therefore, muscle fatigue represents a clinically relevant endpoint that merits rigorous preclinical evaluation. To the best of our knowledge, RyR1‐SERCA1 MTDs have not been investigated in animal studies or clinical trials.

**FIGURE 3:**
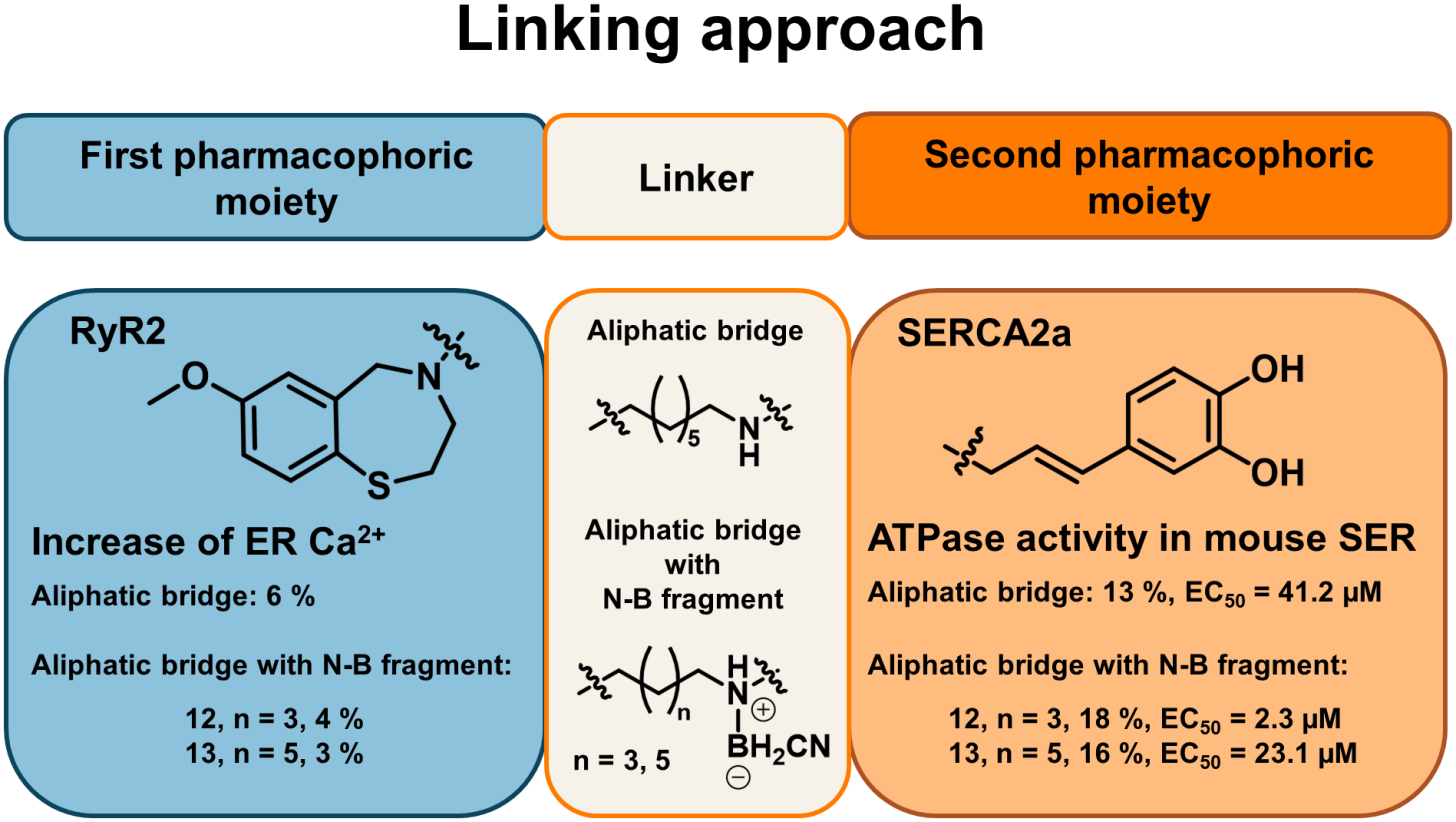
Molecular hybridization with noncleavable linkers. RyR2/SERCA2 dual‐acting compounds were synthesized by conjugating a 1,4‐benzothiazepine scaffold, which functions as an RyR2 stabilizer, to a caffeic acid moiety reported to bind directly to SERCA. Compounds with polar N‐B moieties (**11**, **12**, and **13**) exhibited particularly pronounced SERCA2 activity [39].

Although the linking strategy has successfully combined COX‐2 and sEH, as well as RyR2 and SERCA2 pharmacophores, into multitarget compounds, this approach faces several pharmacological and physicochemical limitations. The resulting molecules often have a high molecular weight and polar surface area, which frequently reduce membrane permeability and compromise bioavailability when administered orally. Because the linked pharmacophores are covalently tethered at a fixed ratio, independent optimization of target coverage is impossible, restricting clinical flexibility across heterogeneous cardiovascular phenotypes. Finally, certain linkers may undergo metabolic degradation or contribute to off‐target effects. Consequently, while linking remains a valuable discovery entry point, its clinical translation requires strategic linker rigidification, bioisosteric replacement, and rigorous ADMET profiling to balance multitarget efficacy and drug‐like properties.

The use of *cleavable linkers* is not a common concept in drug development for CVDs. Nevertheless, dual‐acting prodrugs (codrugs) have recently been developed [53]. It simultaneously modulates the renin–angiotensin‐aldosterone (RAA) and natriuretic peptide (NP) pathways via angiotensin II type 1 receptor (AT1) antagonism and metalloprotease neprilysin (NEP) inhibition and synergistically promotes hemodynamic effects. Based on the structure of the carboxylic acid active metabolite of losartan E‐3174 (Figure 4) and the selected monocarboxylic acid NEP inhibitors [54], the authors synthesized dual structures in which the pharmacophores were linked via an ester linker of different lengths and compositions (OCH_2_O, OCH(CH_3_)O, and OCH_2_CH_2_O). After absorption in the gastrointestinal (GI) tract, such molecules can be hydrolyzed to active carboxylic acids (Figure 4) [53]. The cleavage of the linkers was proven in vitro using human liver and intestine S9 fractions (HLS9 and HIS9, respectively) with a maximum conversion factor to NEP (30.3%) antagonists CHEMBL225085 [54] and E‐3174 (43.1%) detected for the compound containing an OCH_2_O bridge (codrug; **b**, Figure 4). The AT1 antagonism of E‐3174 was determined in previous studies using rat adrenal cortical membranes with an IC_50_ value of 37 nM [55]. The human NEP activity of CHEMBL225085 is 0.9 nM [54]. Antihypertensive action of codrug **3b** was assessed in spontaneously hypertensive rats, which mimic pathological progressive alterations that are present in human hypertension, showed statistically significant reduction in blood pressure‐related parameters (systolic and diastolic blood pressure and heart rate), which were significantly lower than those observed in rodents receiving either losartan alone or losartan in combination with CHEMBL225085 [53]. The authors also noted that codrug **3b** offers advantages not only over losartan but also over E‐317 because E‐3174 would be poorly absorbed if it were administered directly.

**FIGURE 4:**
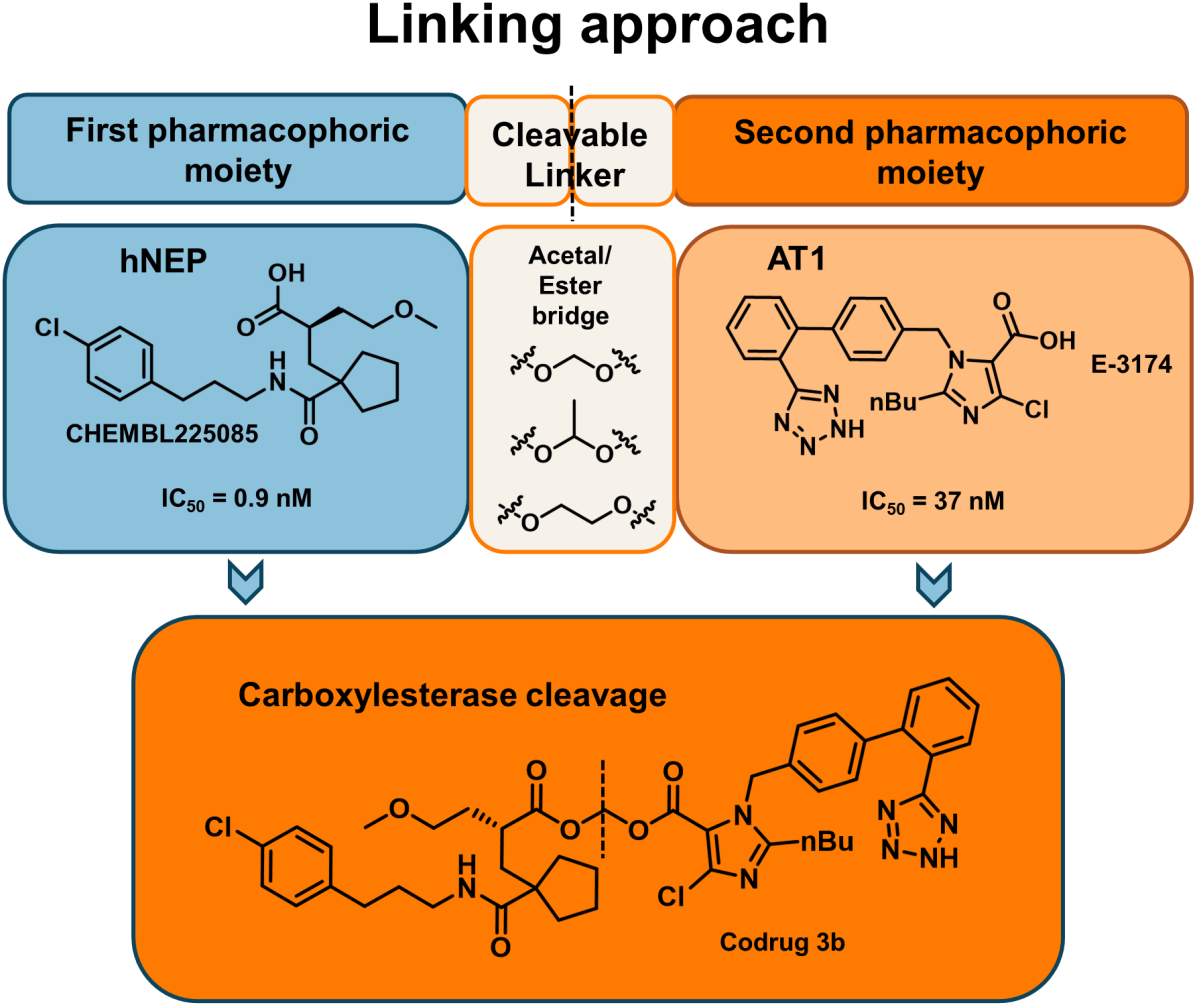
Molecular hybridization with cleavable linkers. The selected NEP inhibitor CHEMBL225085 and the AT1 receptor antagonist E‐3174 were linked via ester linkers [53]. These molecules can be hydrolyzed to active carboxylic acids by carboxylesterases in the liver. Compound **3b**, which contains an OCH_2_O bridge, exhibited the highest conversion efficiency to the AT1 antagonist (43.1%) and NEP inhibitor (30.3%).

The main challenge of the cleavable linker approach is to strike a balance between in vivo stability and kinetics of site‐specific cleavage. Premature linker degradation risks off‐target toxicity, whereas delayed cleavage prolongs therapeutic onset and compromises simultaneous dual‐target engagement. Furthermore, cleavage efficiency is highly dependent on local microenvironmental triggers (e.g., enzyme expression, pH, and redox potential), which exhibit substantial interpatient and diseasestate variability, resulting in unpredictable drug release and inconsistent efficacy.

Overall, the linker approach demonstrates that in the design of hybrid agents, not only the selected pharmacophores but also the linker itself—its length, structure, and position relative to the parent structures—plays a significant role in determining the properties of hybrid molecules.

### 2.2 Fusion Approach

Carocci et al. synthesized analogs of mexiletine, a sodium channel (Na_V_1.x) blocker, by directly fusing it with a 2,2,5,5‐tetramethylpyrroline fragment, which is a free radical scavenger [56]. The goal was to develop dual‐action drugs that could potentially protect the heart from postinfarction reperfusion injury (Figure 5). The antiarrhythmic and antioxidant properties were tested in vitro using isolated guinea‐pig atria to evaluate their inotropic and/or chronotropic effects. The authors induced atrial arrhythmias by applying a sinusoidal 50 Hz alternating current of increases amplitude to the isolated left atrium, paced at 1 Hz [56]. Exposure to hybrid compounds increases the threshold for the onset of ac‐arrhythmia associated with arrhythmogenic cardiomyopathy. The compounds exhibited higher activity than the parent mexiletine, with the EC_50_ value for the most promising tetramethylpyrroline compound analog with a *tert*‐butyl substituent falling within the nanomolar range (Figure 5, compound **2b** R =  *t‐*Bu, EC_50_ = 54 nM). Among all the compounds studied (R = CH_3_, *t‐*Bu, Ph, *i‐*Pr, and Bn), **2b** exhibited the highest antiarrhythmic activity and best cardiovascular profile. Within the series assessed, this compound exhibited the highest proportion of sp^3^‐hybridized carbons. Together with the presence of a stereogenic center, this property is generally associated with greater clinical success as out‐of‐plane substituents can enhance receptor–ligand complementarity [56, 57]. Additionally, the authors tested the compounds on HEK293 cells transiently transfected with human Na_V_1.5 using the whole‐cell patch‐clamp method, and the IC_50_ values were found to be similar for mexiletine and **2b**. The in vitro antioxidant activity was tested using the oxidation of 2,7‐dichlorodihydrofluorescein (DCFH) assay on human hepatocellular liver carcinoma (HepG2) cells. Compounds containing a 2,6‐dimethylphenoxy moiety significantly reduced the H_2_O_2_‐induced oxidation. Hydroxyl radical scavenging was assessed via nonfluorometric hydroxylation of benzoic acid. The tested compounds achieved IC_50_ values of approximately 70 μM, slightly outperforming mexiletine (IC_50_ = 93 μM) [56].

**FIGURE 5:**
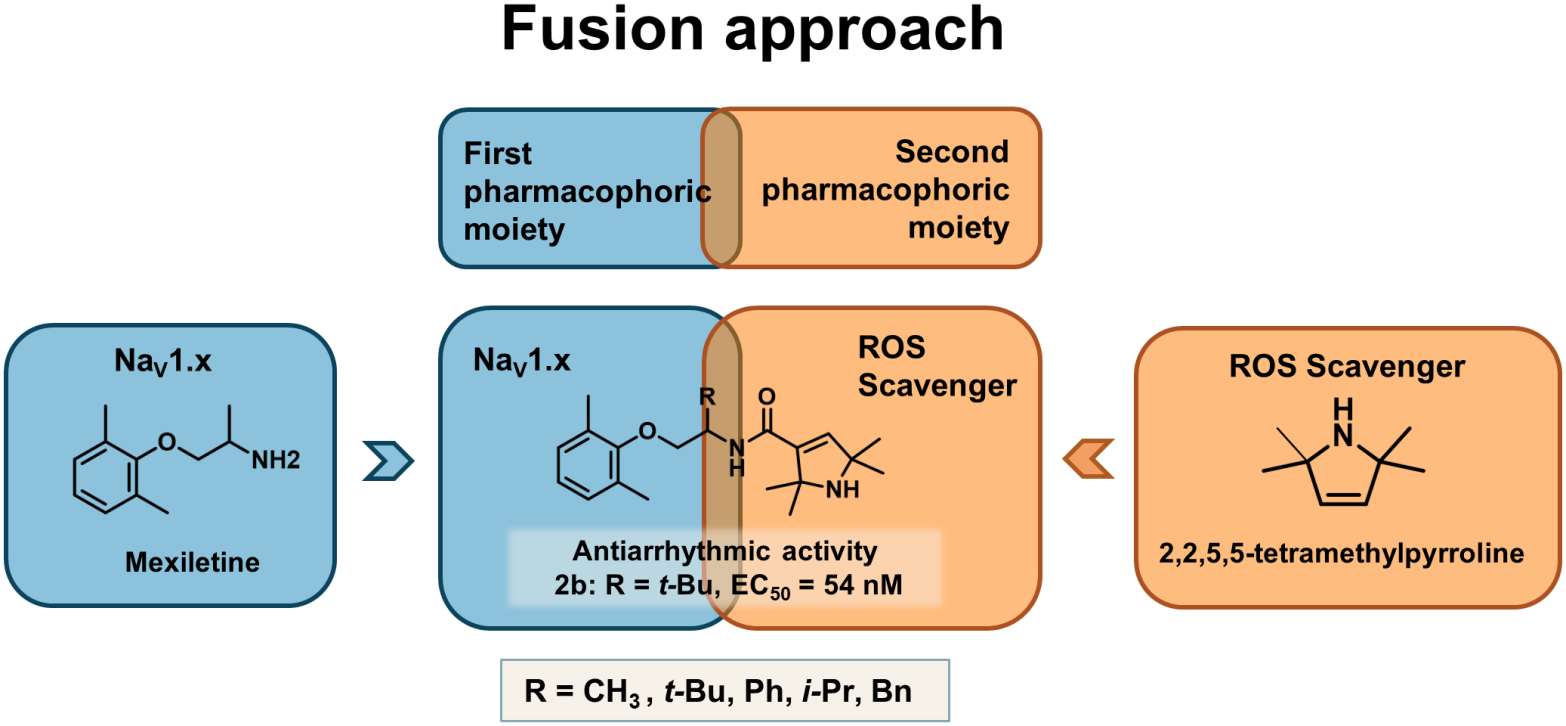
Fusion approach. Antiarrhythmic dual‐action drugs based on the sodium channel blocker mexiletine and ROS scavenger 2,2,5,5‐tetramethylpyrroline [56]. The *tert*‐butyl‐substituted tetramethylpyrroline (**2b**), which was the most potent derivative in this series, displayed greater antiarrhythmic activity than mexiletine and exhibited significant antioxidant effects.

The fusion of established cardiovascular agents with radical scavengers represents a conceptually compelling approach to multitarget drug design, particularly given the central role of redox dysregulation in cardiac pathophysiology. Many critical ion transporters and signaling proteins contain redox‐sensitive residues that are highly susceptible to oxidative modification. A prominent example is RyR2, whose solvent‐exposed cysteine residues undergo S‐nitrosylation, S‐glutathionylation, or direct oxidation under elevated reactive oxygen species (ROS) conditions. These post‐translational modifications promote pathological gain‐of‐function states. By covalently integrating a radical‐scavenging moiety into a cardiologically active scaffold, the resulting hybrid molecule can simultaneously modulate the target channel activity and locally mitigate oxidative stress at the site of action.

The molecular fusion strategy has been widely applied to 1,4‐dihydropyridine (DHP) scaffolds to generate derivatives with expanded pharmacological profiles. DHPs are classically recognized for their pharmacological activity as L‐type calcium channel (Ca_V_1.x) ligands, which are cornerstones in the management of hypertension and ischemic cardiac disorders, such as angina pectoris [58, 59, 60]. DHPs bind to specific receptor sites on Ca_V_1.x, having defined structure–activity relationships [61]. According to the updated binding‐site nomenclature, DHPs bind to the Ca_V_1.2 F3 site [62, 63]. The antihypertensive agent benidipine serves as a well‐established example [64]. Recent fusion‐based designs have endowed these compounds with additional therapeutic properties. For example, several DHP hybrids exhibit multitarget properties, including 11β‐hydroxysteroid dehydrogenase type 1 (11β‐HSD1) inhibition and nuclear factor erythroid 2‐related factor 2 (Nrf2) pathway activation, among others [65, 66]. Pachón‐Angona et al. introduced a one‐pot and three‐component Hantzsch reaction for the synthesis of multifunctional DHPs with propargylamide residues, which are known to enhance nuclear translocation of Nrf2 and its binding to the antioxidant response element (ARE) [66]. The final molecules **4i**–**4l** (Figure 6) exhibited calcium channel antagonistic activity, antioxidant properties, and the ability to activate endogenous Nrf2 antioxidant pathways [66]. At a concentration of 10 μM, these compounds demonstrated the ability to block Ca^2+^ channels in SH‐SY5Y cells (up to 19%, Figure 6). Nrf2 induction potency was determined using a luciferase assay in the AREc32 cell line. All synthesized DHP derivatives containing propargylamine and N‐CH_3_ moieties exhibited CD values (concentration required to double the specific activity of the luciferase reporter) ranging from 69 to 96 μM, comparable to that of melatonin (66 µM). Melatonin is a potent inducer of the Nrf2‐mediated antioxidant response pathway and exerts cardioprotective effects in acute myocardial infarction and ischemia/reperfusion injury through AMPK/Nrf2 signaling [67, 68]. Compound **4b** containing an NO_2_ group in the phenyl ring in the ortho position relative to DHP (Figure 6, R^2 ^ =  NO_2_, R^1 ^ =  R^3 ^= H) activates Nrf2 but has no effect on calcium channels, underscoring the importance of N‐CH_3_ groups for ensuring the dual Ca_V_1.x/Nrf2 activity [66]. Two of the compounds also inhibited cathepsin S (Cat S)—a lysosomal cysteine protease that plays a key role in the breakdown of proteins and antigens within lysosomes (Figure 6; compound **4i** with R^1 ^ =  CH_3_, R^2 ^ =  Cl, R^3 ^ =  H and compound **4h** with R^1^ = R^2^ = H, R^3^ = Br). Among the evaluated compounds, **4i** exhibited the most favorable pharmacological profile, characterized by potent antioxidant activity and significant activation of the Nrf2‐ARE signaling pathway. Furthermore, **4i** weakly inhibited Cat S (*K*_*i*_ = 69 µM) and moderately blocked Ca^2+^ channels, with an efficacy of approximately one‐third that of the reference agent nimodipine.

**FIGURE 6:**
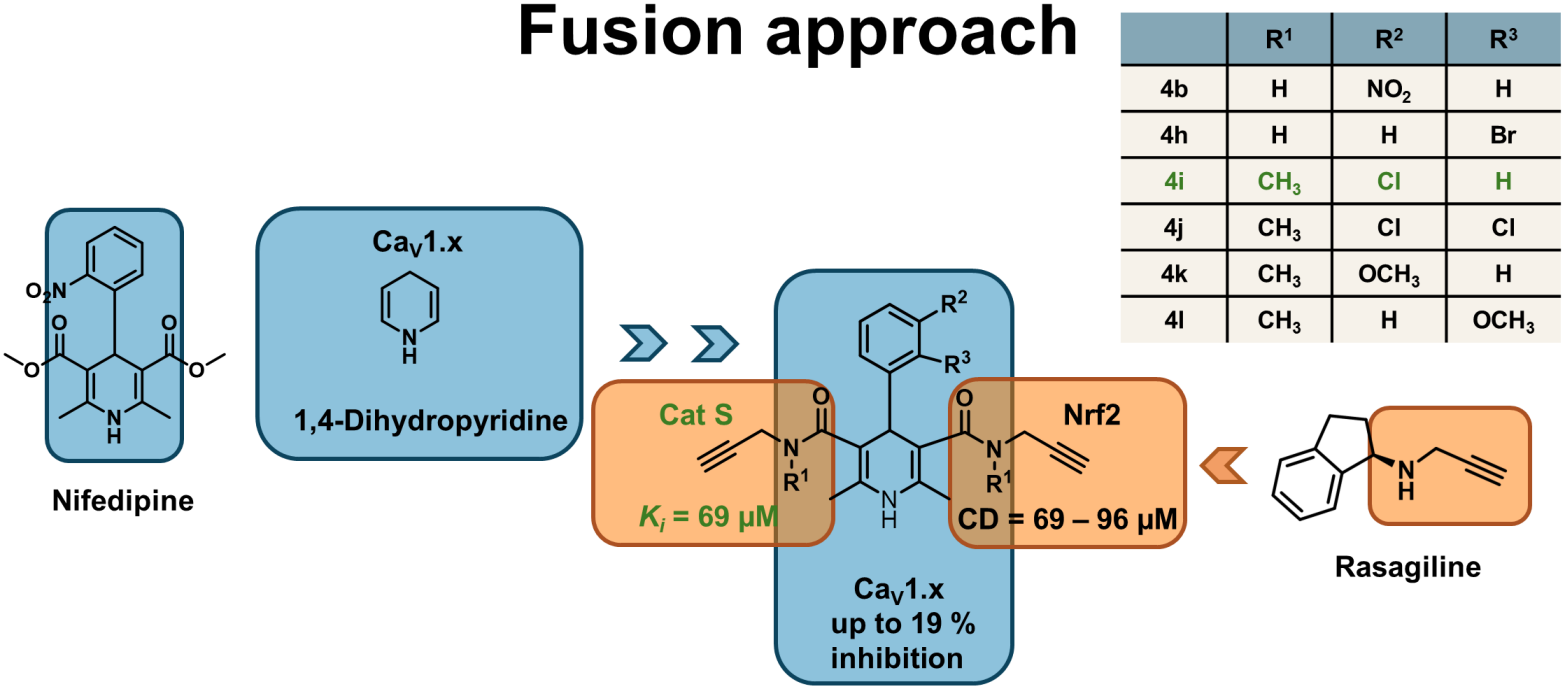
Fusion approach. Development of 1,4‐dihydropyridine derivatives with propargylamide residues [66]. Compound **4i**, the most potent derivative in this series, exhibited a multifaceted pharmacological profile. It blocks L‐type calcium channels (Ca_V_1.x), activates the Nrf2 signaling pathway, inhibits cathepsin S (Cat S), and exhibits significant antioxidant activity.

The authors aimed to develop a convenient synthetic method for producing MTDs for the treatment of Alzheimer’s disease (AD). However, the data suggest that impaired Nrf2 activation is associated with the progression of cardiovascular disease [69]. In this respect, the synthetic approach proposed by Pachón‐Angona et al. can be easily extended to obtain DHPs with Nrf2 activation abilities for cardiological applications. Initially, 1,4‐DHPs were developed as Ca_V_1.x blockers for cardiovascular applications. However, successive studies have shown that they can interact with various biological targets, including subtypes of calcium channels, other channels such as potassium and sodium, and certain G‐protein‐coupled receptors [70, 71, 72, 73]. Therefore, the selective optimization of the side activity of DHPs, which will be discussed below, provides broad possibilities for the development of new multitargeted drugs.

The molecular fusion approach offers the theoretical advantage of simultaneous dual‐target engagement without the entropic penalties associated with flexible linkers. However, this strategy faces significant constraints in the design of cardiovascular drugs. The fusion of distinct pharmacophores often necessitates structural compromise, resulting in reduced affinity for one or both targets and unpredictable selectivity of the resulting compound. Fused scaffolds frequently exhibit suboptimal physicochemical properties, including poor aqueous solubility, limited membrane permeability, and metabolic instability, which compromise their oral bioavailability and cardiac tissue distribution.

### 2.3 Merging of Two Pharmacophores

Molecular merging is a sophisticated strategy for multitarget drug design, typically yielding ligands with superior efficacy and lower molecular weights than those generated by linking or fusing approaches. However, it remains one of the most technically challenging and high‐risk methods in the field [20]. In the merging strategy, key binding features for the chosen targets are integrated into a single compact scaffold (Figure 7). Computing, artificial intelligence (AI) methods, and in silico modeling are actively used to develop such compounds.

**FIGURE 7:**
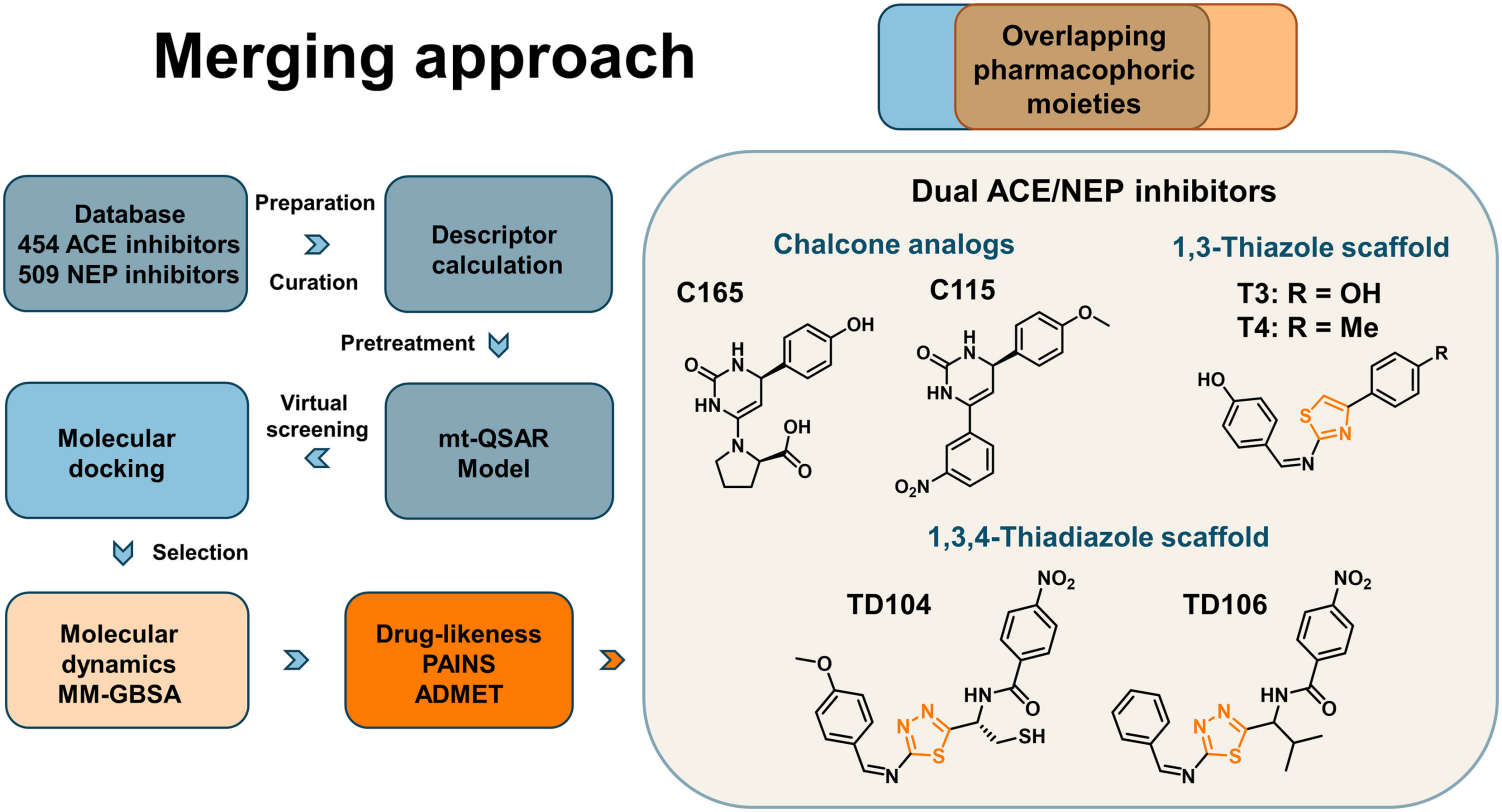
Merging approach. A multitarget QSAR model was coupled with virtual screening to identify potential dual ACE–NEP inhibitors [74]. Computer‐based prediction revealed favorable pharmacokinetic and safety profiles for chalcones **C165** and **C115**, 1,3‐thiazoles **T3** and **T4**, and 1,3,4‐thiadiazoles **TD104** and **TD106**.

In Silico and computational methods utilize molecular simulations, virtual screening, and machine learning to rationally design and predict compounds with multitarget activities against cardiovascular drug targets, optimizing pharmacodynamics and pharmacokinetics. Shah et al. introduced multitarget quantitative structure–activity relationship (mt‐QSAR) model for dual endpoint angiotensin converting enzyme (ACE) and neprilysin (NEP) inhibition prediction [74]. ACE and NEP are central enzymes that regulate blood pressure and cardiovascular homeostasis [74]. Their dual inhibition is clinically attractive for treating conditions such as hypertension and heart failure. The authors applied both linear (genetic algorithm‐linear discriminant analysis) and nonlinear (random forest) machine learning methods. The analyzed dataset included approximately 1000 ACE and NEP inhibitors, which were virtually screened to identify novel dual inhibitors. The selected hits were then examined by molecular docking and molecular dynamics simulations to analyze how they bind to the ACE and NEP active sites. Extensive computer‐based ADME/toxicity studies and analysis of the drug activity of preselected molecules identified several chalcone, 1,3‐thiazoles, and 1,3,4‐thiadiazole derivatives as potential dual ACE‐NEP inhibitors (compounds **C165**, **C115**, **T3**, **T4**, **TD104,** and **TD106**, Figure 7) [74]. Although the study applied standard filters for drug‐likeness, pan‐assay interference compounds (PAINS), and absorption, distribution, metabolism, excretion, and toxicity (ADMET) properties, it did not address synthetic accessibility or provide experimental data to validate the predicted dual‐target activity.

Recent advances in AI are believed to significantly accelerate the discovery and systematic optimization of multitarget agents [75]. The implementation of AI Generative Deep Learning Models enables *de novo* design of molecules tailored for multitarget activities, balancing pharmacodynamic effects, and reducing trial‐and‐error in synthesis and testing [76, 77]. Recent advances include the use of chemical language models (CLM), which generate novel dual ligands by learning and fusing/merging pharmacophore elements from known ligands. Such AI‐driven design enables the discovery of multitarget compounds with balanced activities, followed by experimental validation [76]. Isigkeit et al. trained CLM to generate dual ligands for six pairs of target proteins comprising the pharmacophore elements of ligands for both targets in one molecule [76]. The targets included peroxisome proliferator‐activated receptor delta (PPARδ) and sEH, which play roles in cardiac function. The CLM model, initially trained on 365,000 molecules from the ChEMBL database of bioactive molecules with drug‐like properties, was further refined using template molecules for target pairs. It can identify complex chemical features from SMILES molecular representations and predict the structures of drug‐like molecules similar to known ligands of the target pairs of interest [76]. For the experimental design, chemical structures, and activities, see Figure 8 [76]. The scaffold widely found in sEH inhibitors contains an aromatic tail and urea group [78]. Merging this scaffold with the 3‐(4‐alkyloxyphenyl)propanoic acid pharmacophore of the PPARδ agonist resulted in three dual PPARδ/sEH ligands (compounds **7**, **8**, and **9**, Figure 8b). These molecules were successfully synthesized, and their activities were experimentally confirmed using Gal4‐hybrid reporter gene assays (PPARδ) and sEH activity assays. The authors emphasized that the development of multitarget ligands using the CLM method is feasible when the structures of molecules representing the targets of interest are closely related to each other. However, it becomes a more complex task when dealing with combinations of targets whose ligands exhibit greater chemical diversity, a situation that also complicates the development of multiple ligands using traditional systematic methods [76, 79].

**FIGURE 8:**
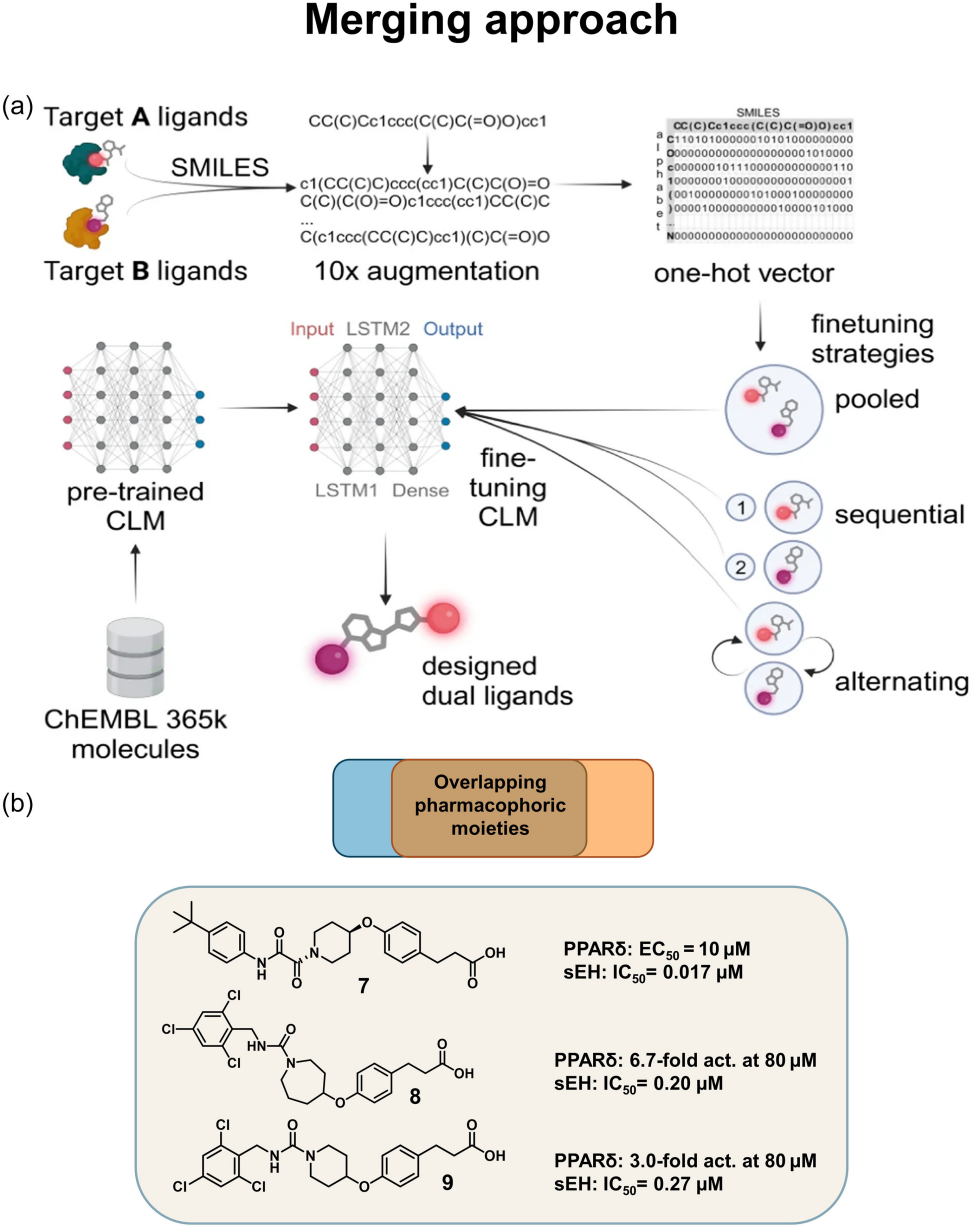
Merging approach. (a) Chemical language models (CLM) for developing multitargeted drugs. (a) Experimental design. String representations, such as SMILES, are used for data training, and new molecules can be designed in a data‐driven fashion. (Reproduced under terms of the CC‐BY license, Ref. [76], Copyright (2024), Laura Isigkeit et al., published by Springer Nature). (b) Chemical structures of PPARδ/sEH ligands and their activities.

Although AI and machine learning have accelerated target identification, scaffold design, and polypharmacological optimization, their implementation in multitarget cardiovascular drug discovery may be limited. Cardiovascular datasets typically originate from heterogeneous sources (clinical trials, EHRs, omics platforms, and imaging) without standardized collection protocols, terminologies, or longitudinal tracking. This fragmentation limits AI model generalization, obscures phenotype‐specific target engagement, and hinders the capture of time‐dependent network dynamics that are essential for multitarget drug design. Additionally, biomarker readouts that directly reflect the state of the targeted protein complexes, such as the downregulation of the Ca_V_1.2 channel within days of AF, are frequently unavailable, hindering the longitudinal monitoring of therapeutic interventions. Addressing these gaps through effective data sharing and management is critical for improving the translational reliability of AI‐predicted cardiovascular therapeutics.

## 3 Polypharmacology and Network Pharmacology

Polypharmacology and network pharmacology are drug design approaches that inherently deal with multiple protein targets involved in the pathophysiology of diseases, exploring the promiscuity of cardiovascular drugs to achieve synergistic therapeutic effects [80]. Camargo‐Ayala et al. developed and created a potential multitarget therapeutic candidate for managing AF, drawing on the common structural characteristics of local anesthetics like lidocaine, ropivacaine, and bupivacaine [81]. This candidate inhibits potassium (K_V_1.5), voltage‐gated sodium channel (Na_V_1.5), and the two‐pore domain K^+^ channel (TASK‐1) (Figure 9) [81]. Taking into account that the selected LA have shared chemical features and are blockers of at least two selected targets, the authors proposed 60 basic core structures for the new molecules. Induced‐fit docking (IFD) simulations of ligand–receptor interactions, combined with binding free energy calculations, enabled the identification of compounds exhibiting the greatest number of interactions and optimal binding free energy values in each channel [81]. The latter was estimated using calculations based on the generalized Born surface area method within the molecular mechanics framework (MMGBSA). Of the 25 most promising compounds with the greatest polypharmacological potential—defined by the highest number of interactions and optimal binding free energy values in each channel—10 compounds were selected for further synthesis. Preliminary evaluations of the synthesized compounds’ inhibitory effects on TASK‐1 and K_V_1.5 were performed using the two‐electrode voltage‐clamp (TEVC) technique on Xenopus laevis oocytes that overexpress these channels [81]. Five compounds were identified during testing and were later evaluated on Na_V_1.5. Compound **6f**, which contains two OCH_3_ groups in the phenyl ring and two *N*‐butyl groups (Figure 9), exhibited the most consistent activity in simultaneously blocking all three channels with IC_50_ values of 21, 0.31, and 82 µM for the Na_V_1.5, TASK‐1, and K_V_1.5 channels, respectively [81]. Further evaluation of the compound was performed using single‐cell patch‐clamp experiments on human atrial CMs isolated from tissue samples of patients undergoing open cardiac surgery for coronary artery bypass grafting or valve replacement. The Na_V_1.5 block caused by **6f** was confirmed by detecting a concentration‐dependent decrease in the action potential amplitude and maximal upstroke velocity only in samples from patients with sinus rhythm. The binding affinity of **6f** for TASK‐1 was confirmed in samples from patients with AF. **6f** significantly increased the duration of the action potential at 50% repolarization. Not all three target parameters were initially considered, and only a clear TASK‐1 effect was observed [81]. The authors proposed compound **6f** as a potentially safer alternative for AF management compared to currently available drugs such as vernakalant, flecainide, and amiodarone. However, whole‐atrial in silico simulations revealed that the cardioversion efficacy of **6f** was only achieved at supratherapeutic concentrations (100 µM) [81].

**FIGURE 9:**
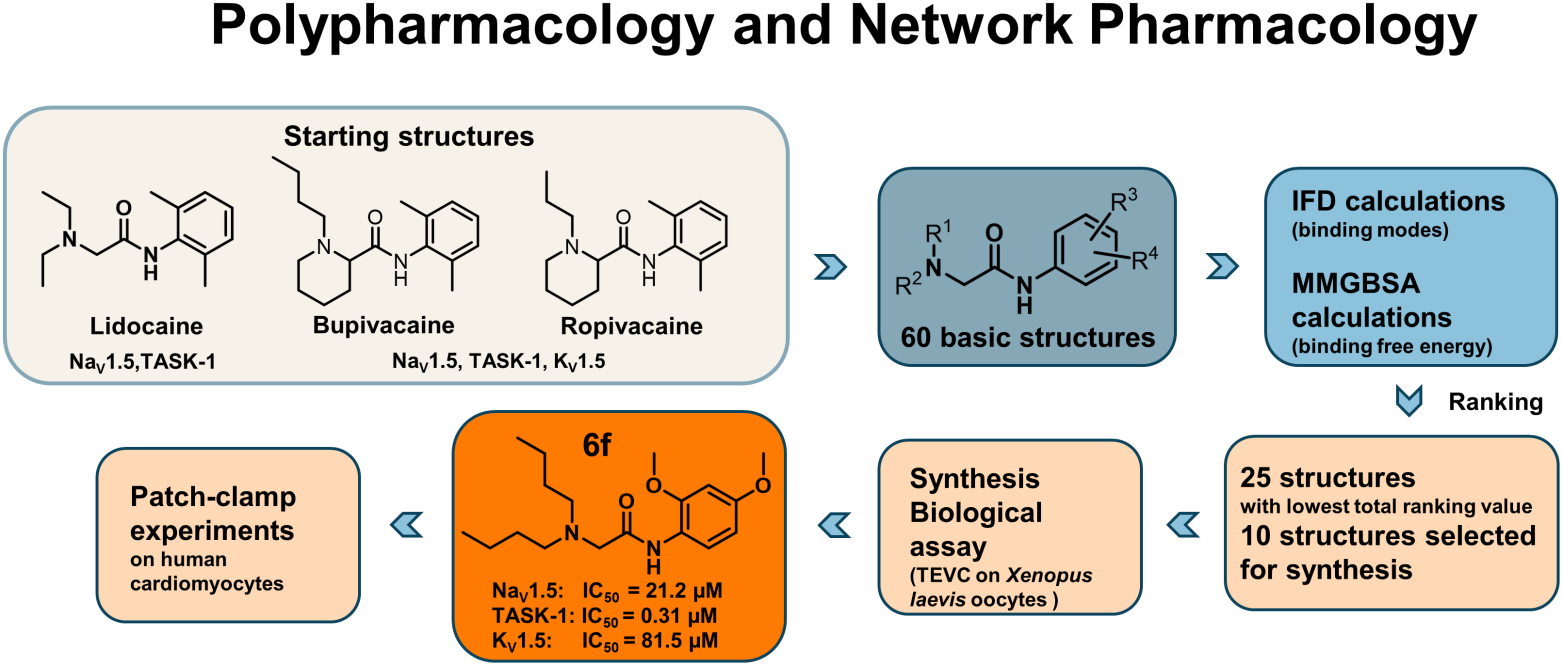
Development of multitarget lidocaine derivative **6f** [81]. The samples were pre‐selected using calculations of binding states and binding free energy, followed by investigation using the two‐electrode voltage‐clamping (TEVC) method. Compound **6f** was the most effective inhibitor of Na_V_1.5, TASK‐1, and K_V_1.5 in this series.

The limitations of polypharmacology and network pharmacology are typically associated with difficulties arising from computational biases inherent in these approaches, issues with the reproducibility of results, and artifacts caused by methodological factors [82]. Molecular docking frequently yields false positives owing to oversimplified scoring and inadequate modeling of protein flexibility and solvent effects. Network propagation is inherently biased toward well‐connected hub proteins, reducing disease specificity and risking circular reasoning in the results.

## 4 Selective Optimization of Side Activity

Another strategy for the development of multipurpose drugs is the selective optimization of side activity (SOSA), which originally stems from a repurposing approach. SOSA changes the basic structure of a substance to improve its side activity while maintaining (enhancing or suppressing) the main target activity [83]. Rational design and consistent modification of the basic structure, followed by screening of the proposed compounds for desired pharmacological properties, have led to the development of new derivatives specifically designed to improve the required side activity. Ong et al. recently applied this approach, creating compounds that are highly selective for intermediate‐conductance Ca^2+^‐activated K^+^ channels (K_Ca_3.1 blockers) and also have a moderate effect on Ca_V_1.x, showing a dual‐target action (Figure 10) [84]. K_Ca_3.1 is expressed in the heart, particularly under pathological conditions such as cardiac fibrosis, remodeling after myocardial infarction, and AF [85, 86, 87]. Some DHP derivatives, including nifedipine, nitrendipine, and nimodipine, exhibit off‐target K_Ca_3.1 activity [70, 88]. Ong et al. applied the SOSA method to modify the structure of DHP compounds, which were initially chosen for their effectiveness against K_Ca_3.1 [84]. The structure–activity relationships revealed that compounds containing a CF_3_ group in the meta‐position relative to DHP inhibited K_Ca_3.1 at nanomolar concentrations (Figure 10) [84]. The authors then compared the blocking abilities of the selected compounds with respect to Ca_V_1.2 and K_Ca_3.1 channels. The authors aimed to diminish the original main Ca_V_1.2 activity and develop a selective K_Ca_3.1 inhibitor. They achieved this goal using a compound containing a fluorine atom in the para‐position relative to DHP and two methyl acetate groups at R^5^ and R^6^ (Figure 10). Compound DHP‐103 blocked K_Ca_3.1 channels with an IC_50_ value of 6.1 nM and a >2000‐fold selectivity over the Ca_V_1.2 channel [84]. We believe that the SOSA approach can also be applied to modify the 1,4‐dihydropyridine skeleton to obtain structures with balanced activity against both Ca_V_1.2 and K_Ca_3.1 and to evaluate their synergistic effects. In this regard, the structure of another compound published by Ong et al. (DHP‐57) with R^7 ^= R^8 ^= CH_3_ and R^6 ^ =  CH_3_COCH_2_ may serve as a starting point. This compound exhibited IC_50_ values of 1.5 and 16.8 nM for Ca_V_1.2 and K_Ca_3.1, respectively (Figure 10).

**FIGURE 10:**
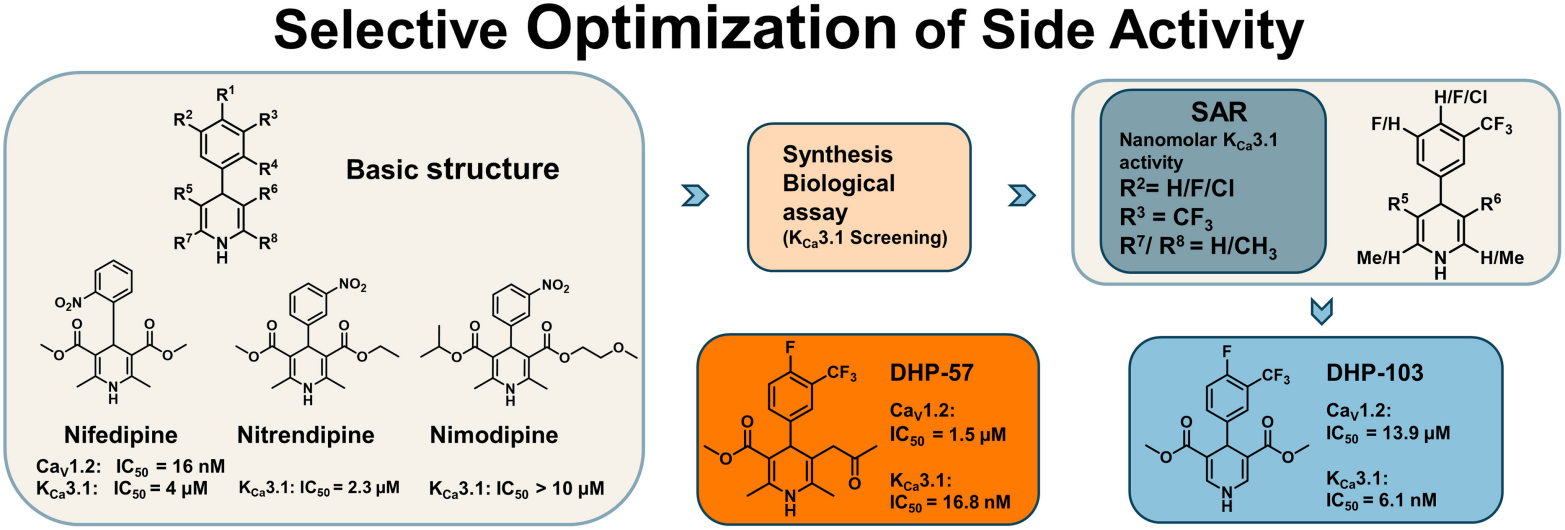
Development of dual Ca_V_1.2/K_Ca_3.1 inhibitors DHP‐57 and DHP‐103 [81]. Structural modification of the dihydropyridine (DHP) core yielded novel derivatives with significantly enhanced K_Ca_3.1 inhibitory activity, converting an initially unintended off‐target effect into a primary pharmacological property.

Wang et al. combined SOSA and molecular hybridization approaches to develop a multitarget α_1_ and 5‐HT_2A_ receptor antagonist (**MT‐1207**), a potential antihypertensive drug (Figure 11) [89]. The authors conducted molecular docking studies based on the crystal structure of the 5‐HT_2A_ receptor and used the structure of 1‐[(2,3‐dimethylphenoxy)propyl]‐4‐(2‐methoxyphenyl)piperazine hydrochloride (**MT‐77**, Figure 11) as a starting point. The latter exhibited α_1_‐adrenoceptor blocking and moderate antagonism of both the 5‐HT_2A_ serotonin receptor and α_2A_/α_2B_‐adrenoceptor functions [90, 91]. The **MT‐77** molecule contains a phenyl A‐ring, an alkyl‐substituted piperazine linker, and a substituted phenyl B‐ring (Figure 11). Based on molecular docking analysis, the authors identified the variable structural elements required to enhance the antagonistic activity of potential 5‐HT_2A_/α_1_ MTDs. Given that *N*‐arylpiperazines or *N*‐arylpiperidines have been described as pharmacophores with affinity for both 5‐HT_2A_ and α_1_ receptors, the authors developed molecules with structures containing these motifs [89]. The synthesized compounds were subsequently selected using radioligand binding assays on Chinese hamster ovary (CHO) cell membranes expressing either recombinant human α_1_‐ adrenoceptor or human 5‐HT_2A_ receptor. A structure–activity relationship (SAR) study showed that the affinity of the compounds increased when a benzoheterocycle was used as ring A and 1*H*‐substituted benzotriazole as ring B. The affinity was influenced by the length of the alkyl chain between arylpiperazine/arylpiperidine and ring B. The derivatives exhibited favorable affinities with *n* = 3 or 2 [89]. The most promising candidate, **MT‐1207**, demonstrated an extremely potent antagonistic effect against the α_1A_ adrenergic receptor (IC_50_ < 0.1 nM) and two other adrenergic receptor subtypes, α_1B_ and α_1D_ (IC_50_ = 0.15 and 1.4 nM, respectively). A pronounced inhibitory effect and high affinity were observed for the 5‐HT_2A_ receptor as well (IC_50_ = 0.27 nM). In vivo studies have demonstrated that **MT‐1207** exhibits superior efficacy compared to single‐target agents, achieving effective blood pressure reduction in a two‐kidney, one‐clip (2K2C) rat model while preserving renal function. The compound is currently undergoing Phase II clinical trials [89].

**FIGURE 11:**
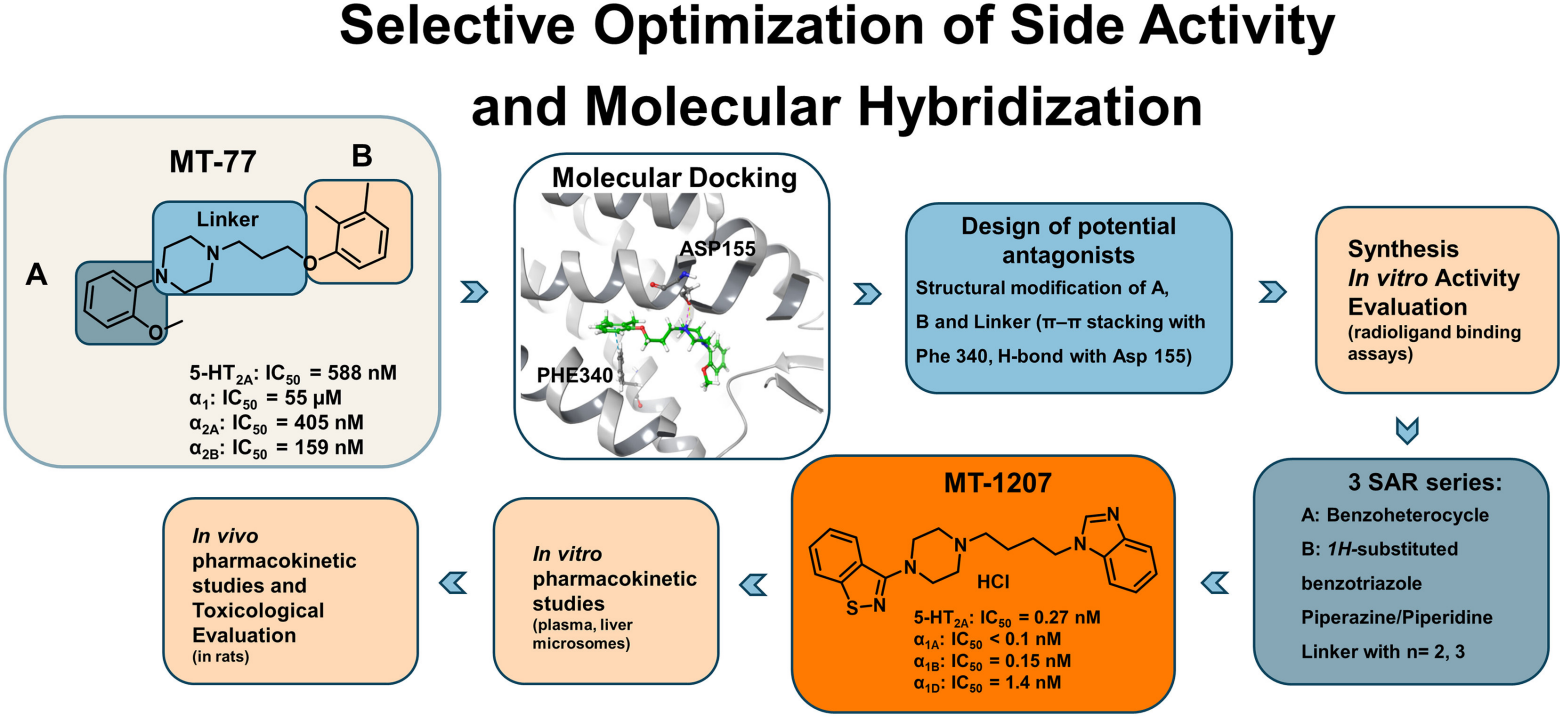
Combinational approach, SOSA and molecular hybridization. The highly potent multitarget compound **MT‐1207** was developed from **MH‐77**, a moderate 5‐HT_2A_ antagonist and α_1_‐, α_2A_‐, α_2B_‐adrenoceptor blocker—through molecular docking‐guided design and three successive series of structure–activity relationship (SAR) studies. Molecular docking is reprinted with permission from Ref. [89]. Copyright (2026), Ong et al., published by American Chemical Society. **MT‐77** IC_50_ Refs. [90, 91].

The SOSA approach offers a distinct advantage in drug discovery by leveraging compounds that have already been administered to humans, thereby providing a stronger basis for assessing their safety and pharmacokinetic behavior. In addition, SOSA can shorten development timelines and reduce costs and attrition of clinical efficacy trials relative to *de novo* discovery or high‐throughput screening while still enabling the identification of novel and patentable lead compounds. However, this approach has several limitations. Because SOSA typically relies on retrospective observations of off‐target activities rather than prospective designs, it lacks the predictive power required for *de novo* multitarget optimization. The simultaneous optimization of multiple pharmacological effects within a single scaffold often introduces highly nonlinear structure–activity relationships, rendering medicinal chemistry iterations unpredictable and increasing the risk of compromising primary target affinity.

## 5 Peptide Therapeutics

Peptide therapeutics represent a promising, yet emerging, class of treatments for heart diseases. However, interest in these compounds and their development is growing [92]. Dwarf open reading frame (DWORF) is a cardiomyocyte‐endogenous small peptide that competes with inhibitory phospholamban (PLN) for binding to SERCA2a Ca^2+^‐ATPase. DWORF overexpression in mice with the pathogenic biallelic PLN‐R14del truncating mutation and rapid‐onset heart failure within weeks improved survival, at least in part, through amelioration of the abnormal clustering of SER Ca^2+^ store organelles [93]. Exogenous peptides are designed to be more selective and have higher specificity for their targets than chemically synthesized compounds. However, their low bioavailability when taken orally and rapid elimination from the body may limit their use [92, 94]. Kehr et al. developed a synthetic, cell‐penetrating **S100A1ct** peptide that improves intracellular Ca^2+^ cycling in CMs and prevents Ca^2+^ imbalance by modulating the activity of SERCA2a and RyR2 [95]. This approach shows promise for the development of new peptide drugs for the treatment of heart failure with reduced ejection fraction (HFrEF) [95]. The authors’ method was based on the natural human Ca^2+^‐binding protein S100A1, which regulates the activity of several key molecular effectors through specific protein interactions, which has recently been solved for the full‐length native skeletal muscle RyR1 Ca^2+^ release channel [96]. For peptide synthesis, amino acid residues 75–94 of the parent S100A1 protein were chosen (Figure 12, the initial three‐dimensional coordinates of S100A1 were retrieved from the Protein Data Bank (PDB ID: 5K89 [97])). These residues correspond to the hydrophobic C‐terminal α‐helix of the S100A1 protein, which interacts with the corresponding molecular epitopes on RyR1, enhancing RyR1‐dependent SR Ca^2+^ release [100, 101]. According to the molecular docking results, S100A1ct tends to form a transmembrane helix and may exert an activating effect on SERCA2a by disrupting the binding of the inhibitory peptide PLB to the transmembrane region of SERCA2a (Figure 12; the structural data used in the figure were adapted from the RCSB PDB under accession codes 6JJU [98] (SERCA2a E1 state) and 2LPF [99] (PLB))[95]. A groove formed by three transmembrane helices, TM2, TM6, and TM, was identified as a possible binding site (Figure 12). SERCA2a activity was tested on SR vesicles prepared from murine S100A1 knockout hearts proving the cardiomyocytes (AVCMs) and in vivo on healthy mice and in postmyocardial infarction models (murine and porcine), showing improved contractile performance and survival in preclinical heart failure models with reduced ejection fraction [95]. The results of organ biomarker analysis, coagulation tests, and basic blood tests conducted on murine and porcine samples revealed no signs of acute or chronic organ damage or dysfunction in response to treatment with **S100A1ct** peptide.

**FIGURE 12:**
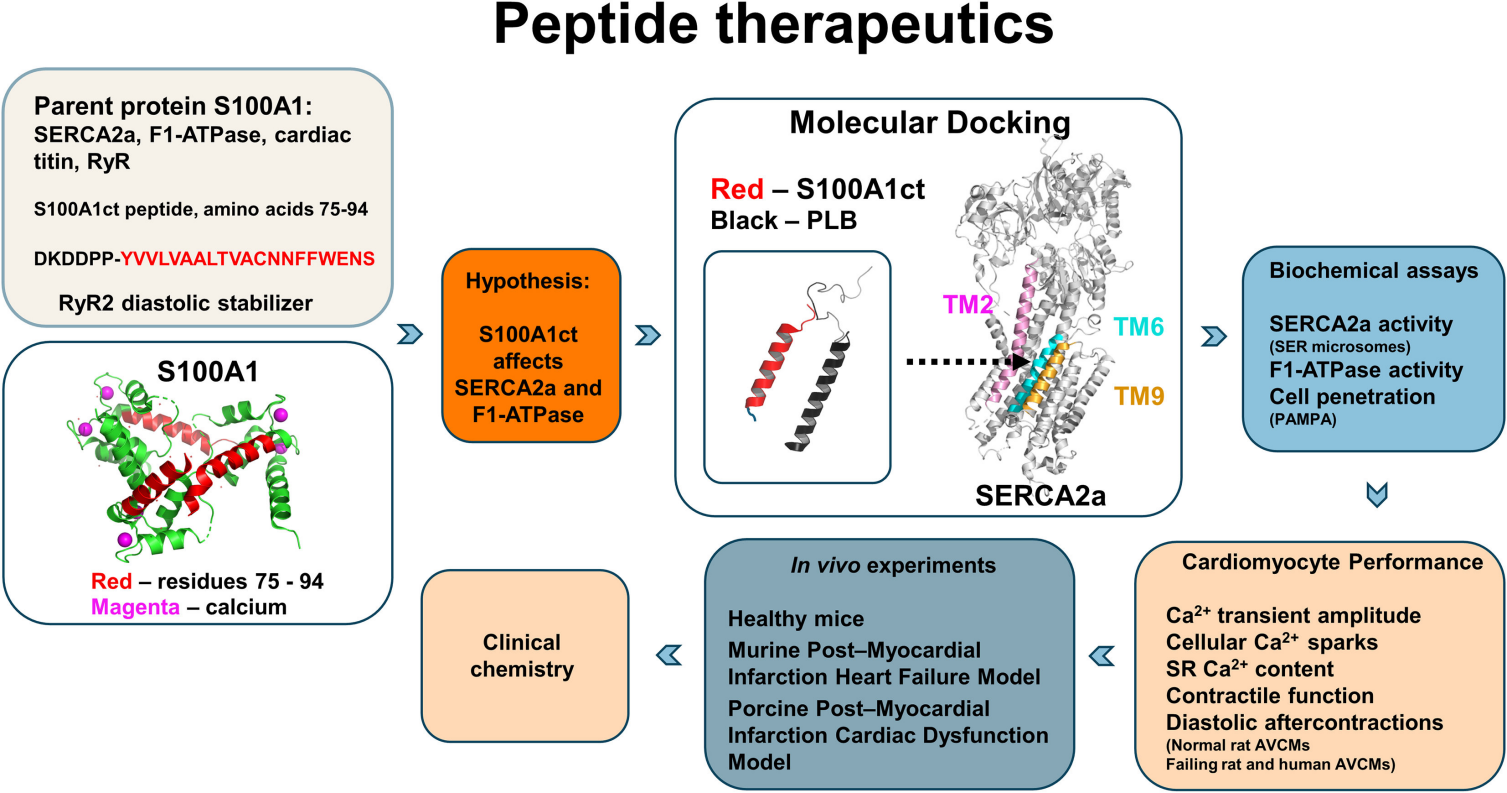
Multitargeted peptide therapeutics. A synthetic, cell‐penetrating peptide, S100A1ct, was engineered from the C‐terminal domain of the cardiomyocyte protein S100A1, which can interact with key effector proteins in cardiomyocytes, such as SERCA2a and RyR2 [95]. The activation of SERCA2a by the peptide may result from competition with phospholamban (PLB) binding to the transmembrane domain of SERCA2a, thereby relieving PLB‐mediated inhibition. The structural models were adapted from the crystal structure of human S100A1, SERCA2a E1, and PLB deposited under PDB accession codes 5K89 [97], 6JJU [98] and 2LPF [99], respectively.

The use of endogenous or exogenous peptide therapeutics is limited compared to conventional cardiovascular drugs because of stability and delivery challenges, particularly in the heart. The abundance of polar amino and carboxyl groups, which confer high hydrophilicity to peptides, also hinders their diffusion through lipid membranes. In addition, peptides are rapidly degraded by digestive enzymes in the GI tract [94]. These issues can be mitigated through a combination of structural modifications (e.g., backbone cyclization, N‐methylation, or D‐amino acid substitution), conjugation strategies, prodrug design, and advanced delivery systems [102].

## 6 Other Approaches: Drug Repurposing, Rational Medicinal Chemistry, Systematic Screening

*Drug repurposing* involves identifying new therapeutic uses for existing approved drugs often by leveraging their multitarget profiles to address complex heart diseases [103, 104, 105]. This approach offers significant advantages over other strategies, as it eliminates the discovery stage, thereby reducing the time and cost of drug development [103]. Known human safety data from prior approvals minimize failure rates in clinical trials, in contrast to new compounds with high attrition [106]. For example, SGLT2 inhibitors (e.g., empagliflozin and dapagliflozin), which were originally developed to treat diabetes, have been used in HFrEF due to their multifaceted effects, including inflammation reduction and cardioprotective benefits [107]. Recently, Ou et al. published a comprehensive review on drug repurposing in CVDs, which covered antidiabetic agents (sodium‐glucose cotransporter‐2 inhibitors, glucagon‐like peptide‐1 receptor agonists, biguanides, thiazolidinediones), diuretics (mineralocorticoid receptor antagonists), antigout agents (colchicine, probenecid), and antiinflammatory/immunomodulatory drugs [104]. Therefore, we will not discuss this topic in detail in the current review.

Using a systematic approach based on the principles of *rational medicinal chemistry*, Gutiérrez‐Hernández et al. designed a series of novel benzimidazoles that exhibited vasorelaxant and antihypertensive efficacies [108]. Building on the structural framework of angiotensin II receptor blockers (ARBs) such as losartan (IC_50_ = 10 nM) [109], candesartan (IC_50_ = 5 nM) [110], valsartan (IC_50_ = 45 nM) [110], and telmisartan (IC_50_ = 9.2 nM) [111], the authors synthesized a series of benzimidazole‐based compounds. These derivatives incorporate a benzimidazole scaffold that functions as a hydrogen bond donor/acceptor within the calcium channel pore recognition site (Figure 13) [108]. The authors performed a pharmacological consensus analysis (PhaCA) [112] to score the predicted pharmacokinetic, pharmacodynamic, biopharmaceutical, and toxicological profiles of the synthesized compounds. Compounds **1** and **5** (Figure 13), which exhibited the highest composite scores, were prioritized for further evaluation. These compounds were tested for their vasodilatory activity in isolated rat aorta. The results showed that compound **1** blocked L‐type calcium channels with an IC_50_ value of 0.57 µM. Subsequent ex vivo experiments investigating ANA II‐induced contraction of the rat aorta and a perfusion study using isolated rat kidneys demonstrated that **1** is an antagonist of type 1 ANA II receptors (Figure 13). The vasorelaxant and antihypertensive effects of the lead compound were demonstrated in vivo in spontaneously hypertensive rat models. Intraperitoneal administration of compound **1** at 20 mg/kg resulted in a 25% reduction in systolic blood pressure [108]. Despite these findings, the authors acknowledged that the in vivo antihypertensive efficacy of compound **1** may not be exclusively driven by the two identified mechanisms as off‐target effects may also play a role.

**FIGURE 13:**
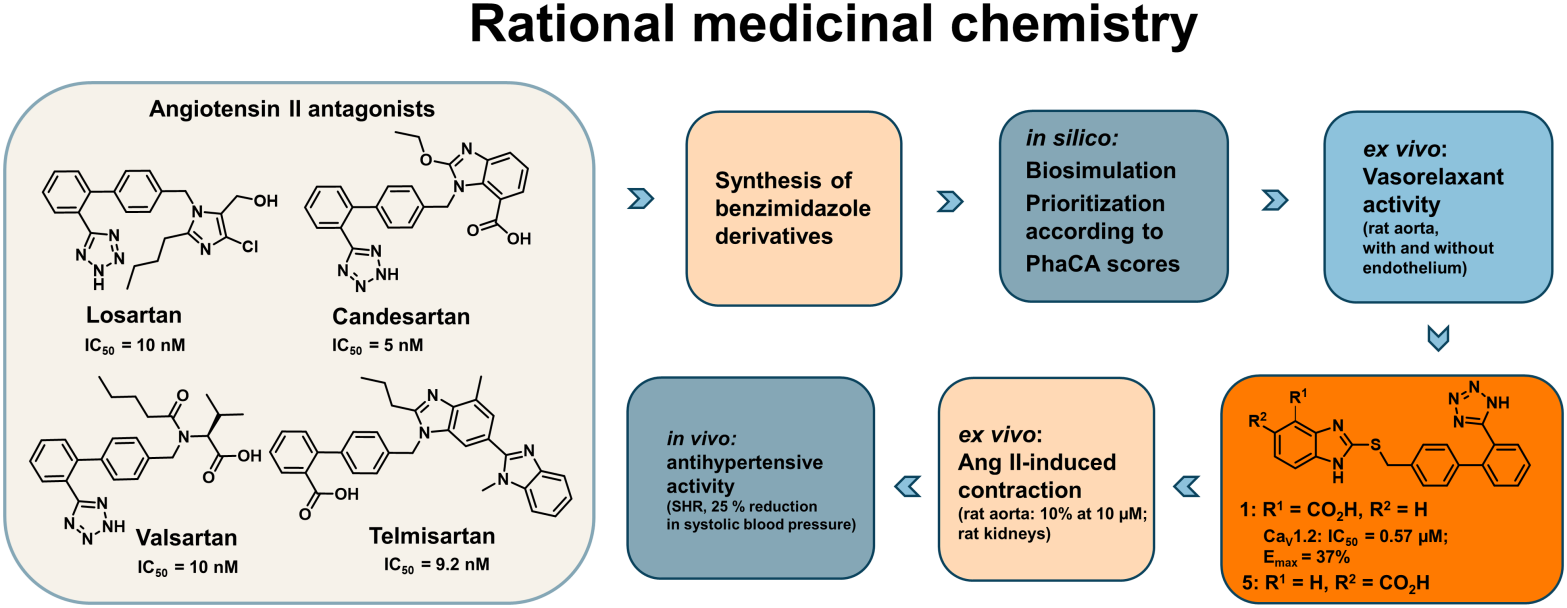
Rational medicinal chemistry approach. Ang‐II/CaV1.x multitarget agents were developed by incorporating a benzimidazole group into the scaffold of angiotensin II receptor blockers. Benzimidazole acts as a hydrogen‐bond donor/acceptor within the binding site of the calcium channel [108]. The IC_50_ of known drugs—Refs. [109, 110, 111].

As *rational drug design* strategy, *bioisosteric replacement* was used by Gündüz et al. to create dual L‐ and T‐type Ca^2+^ channel blockers [113]. The dual L‐ and T‐type Ca^2+^ channel blockade regulates the calcium oscillations necessary for adrenal aldosterone synthesis, which impacts blood pressure regulation [114]. Bladen et al. created 1,4‐DHPs with cyclohexane rings fused to the dihydropyridine ring, which demonstrated efficient blocking of L‐ and T‐type channels (compounds **M2** **–M4**, Figure 14) [115]. Later, dual equipotent inhibition of Ca_V_1.2 and Ca_V_3.2 was detected in Biginelli dihydropyrimidines (DHPM), which are structurally very similar to 1,4‐DHPs (Figure 14) [113]. Swapping of the 1,4‐DHP core for a DHPM scaffold in Ca^2+^ channel blocker design was driven by the need to enhance chemical stability. Specifically, 1,4‐DHPs are prone to photodegradation and oxidative conversion to pyridine derivatives, which make the 1,4‐DHP formulation and postadministration complicated. Replacing the 1,4‐DHP core with a DHPM moiety yields molecules that are less light‐sensitive and more resistant to oxidation [113]. Furthermore, separation and testing of the two enantiomers for their activity toward Ca_V_1.2 and Ca_V_3.2 showed that the *R*‐enantiomer was responsible for the inhibition of both calcium channels (compounds **GD4** and **GD10**, Figure 14) [113]. The authors synthesized and evaluated DHPM analogs of nifedipine, a first‐generation calcium channel blocker. Investigating the influence of this bioisosteric replacement on the pharmacological profile of advanced calcium channel blockers could provide valuable structure–activity insights. For instance, converting clinidipine, a dihydropyridine with dual L‐ and N‐type calcium channel‐blocking activity, into a structurally analogous DHPM derivative may yield compounds with enhanced chemical and photostability, improved metabolic resistance, and potentially refined target selectivity. These modifications could translate to more predictable pharmacokinetics, simplified formulations and storage requirements, extended shelf life, and reduced risk of light‐induced degradation, ultimately strengthening the clinical viability of multitarget calcium channel modulators.

**FIGURE 14:**
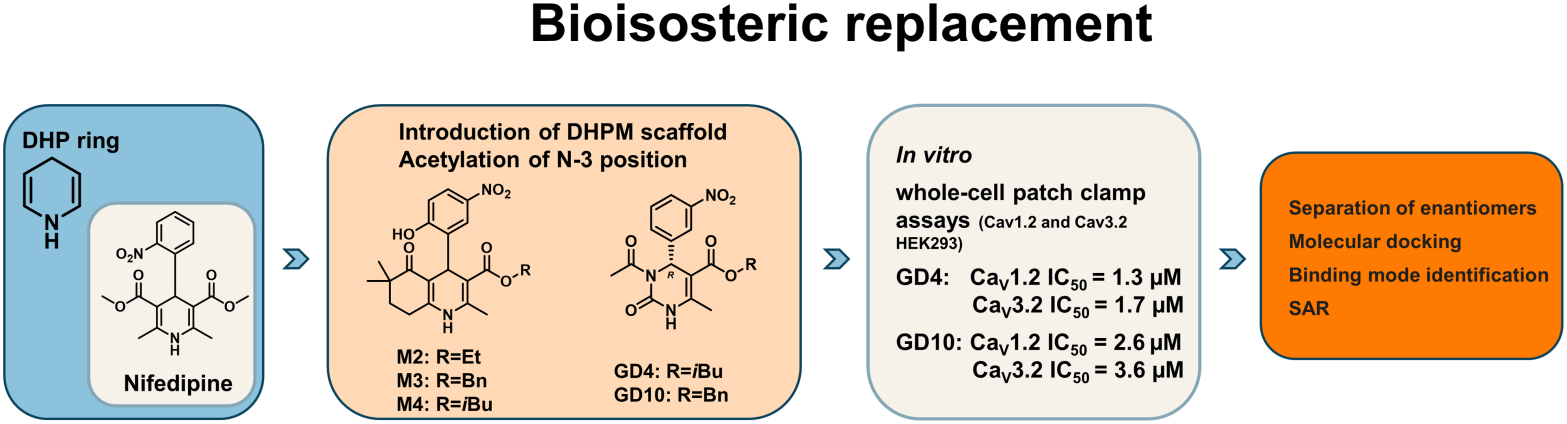
Development of dual L‐ and T‐type Ca^2+^ channel blockers using a bioisosteric replacement strategy [113, 115]. Swapping the 1,4‐DHP core with the DHPM scaffold in Ca^2+^ channel blocker design yields molecules with enhanced chemical stability.

In our recent studies, we implemented a systematic screening approach to develop dual RyR2/SERCA2a agents based on 1,4‐benzothiazepines [116, 117]. We investigated the effects of the synthesized derivatives on intracellular Ca^2+^ signals in a plate reader assay using HEK‐293‐RyR2‐R‐CEPIA1*er* cells (Figure 15) [118, 119]. By investigating the effects of these compounds on Ca^2+^ saturation in the SER of HL‐1 cardiac cells and subsequently on their ability to enhance SERCA2a‐mediated ATP hydrolysis in cardiac microsomes, we identified compounds with a dual effect: they prevent Ca^2+^ leakage through RyR2 and increase Ca^2+^ saturation in the SER by activating SERCA2a [116]. Of particular note are 1,4‐benzothiazepines and their structural analogs containing cyclopropanol groups. We have shown that relatively minor structural changes in their structures lead to differences in their dual properties with respect to RyR2 and SERCA2a. This distinctive feature enables the development of drugs that can be adapted to individual needs and used in future studies to create dual‐action agents designed for personalized treatments. Our lead compound, GM1869, a 1,4‐benzothiazepine with two cyclopropanol groups linked via an aliphatic linker, exhibited an EC_50_ value of 2.7 µM in HEK‐293‐RyR2‐R‐CEPIA1*er* cells and a value of 383 nM in mouse SER microsomes [116]. The compound also enhanced Ca^2+^ uptake in human and murine CMs, the latter from WT and RyR2R2474S/+ mouse hearts [117]. Investigations on human iPSC‐CMs showed that GM1869 significantly delayed the rise time and accelerated the decay time of Ca^2+^ transients, indicating that this compound affects the intracellular human‐like RyR2 and SERCA2a functions. The estimated IC_50_ values for the rise times were 25 pM (WT) and 45 pM (heterozygous pathogenic RyR2 mutation R2474S+/−). The EC_50_ values for the decay times were 97 pM (WT) and 71 pM (R2474S+/−) [117]. Future studies should investigate the stimulatory versus inhibitory effects of post‐translational modifications on the allosteric dimeric native SERCA2a and tetrameric RyR2 protein complexes in the context of dual‐inhibitory MTDs in CMs and cardiac tissues.

**FIGURE 15:**
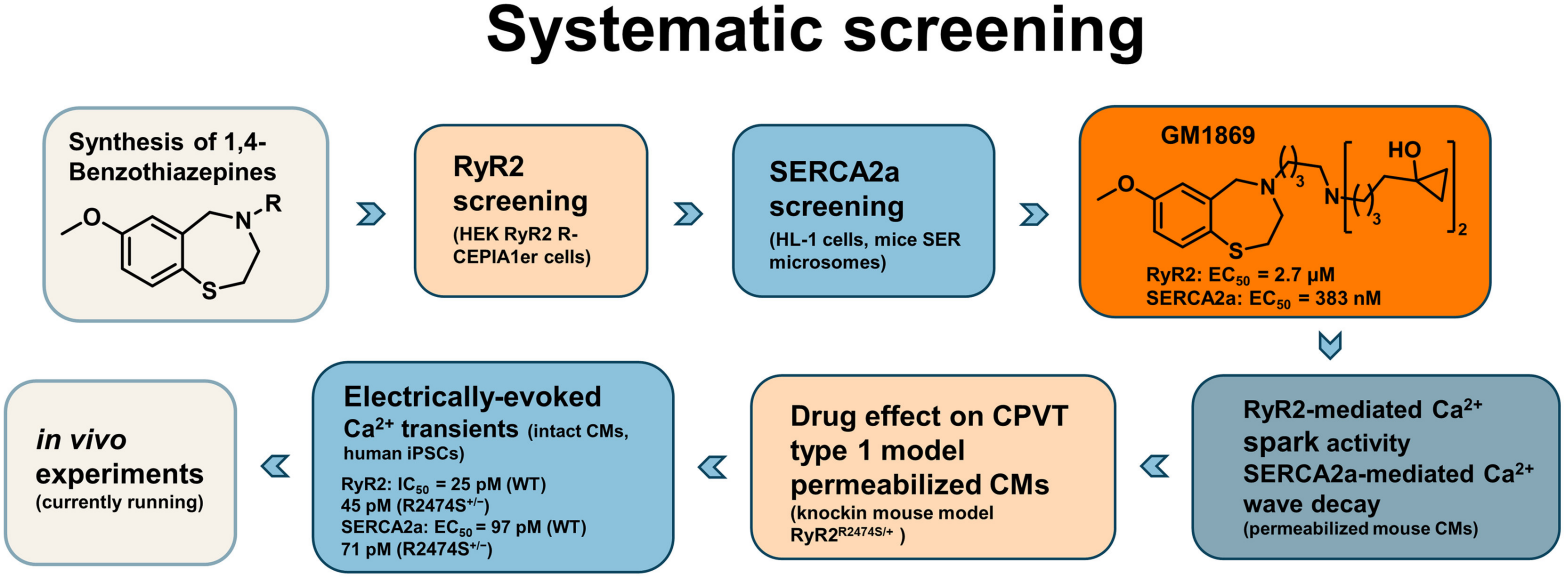
Screening approach. Development of RyR2/SERCA2a agents [116, 117]. Several synthetic compounds based on 1,4‐benzothiazepines have been investigated for their ability to stabilize RyR2 and activate SERCA2a. The results showed that the cyclopropanol derivatives exhibited this dual effect, with GM1869 demonstrating the greatest potency in this series.

Zhang et al. systematically screened a series of indole‐substituted acetamide derivatives [120]. The identification of the molecular target resulted in a novel MD2/ADAM17 dual‐target inhibitor as a potential therapeutic candidate for saturated fatty acid‐induced myocardial inflammatory injury [120]. Based on a screening of Raw264.7 cells, compound **#3** was identified as a substance that suppresses the production of interleukin‐6 (IL‐6) and moderately inhibits tumor necrosis factor alpha (TNF‐α) (Figure 16). These two factors impair cardiomyocyte function, contributing to cell death, fibrotic remodeling, and ultimately cardiac dysfunction [121, 122]. Because compound **#3** selectively blocked the Nuclear Factor‐kappa B (NF‐κB) pathway with no effect on MAPK signaling, the authors suggested that its antiinflammatory mechanism may not fully depend on the canonical signaling cascade in the innate immune system [120]. Studies on the cardiovascular function of compound **#3** in obese mice induced by a high‐fat diet (HFD) showed a significant reduction in systolic, mean, and diastolic blood pressure, with no detectable effect on diet‐induced hyperglycemia or dyslipidemia. Transcriptomic analysis established a link between compound **#3** and the IL‐17, TNF, and toll‐like receptor (TLR) signaling pathways [120]. Reverse docking, surface plasmon resonance (SPR), and cellular thermal shift assay (CETSA) were used to identify ADAM17 as the molecular target (Figure 16). Compound **#3** binds to ADAM17 with *K*_*d*_ = 0.634 µM. The binding site, which was found to be distinct from the conserved zinc‐binding motif, was determined using protein conservation analysis, molecular docking, and CETSA on ADAM17 mutants derived from H9C2 cells. Because **#3** markedly decreased the abundance of mature protein, the authors suggested that its pharmacological mechanism may extend beyond ADAM17 targeting [120]. Multiple methodologies were applied to identify the second target of **#3**. The authors performed in vitro kinase assays and found that **#3** suppressed PA‐induced activation of P38MAPK, a key MAPK pathway protein [120]. The P38MAPK expression levels were reduced in ADAM17‐knocked‐down H9C2 cells, suggesting that compound **#3** may act on upstream MAPK regulatory proteins. Investigations of MD2 knockdown in H9C2 cells showed reduced levels of P‐P38MAPK, suggesting that compound **#3** binds to MD2. This assumption was confirmed by SPR using the immobilized MD2 protein, yielding a *K*_*d*_ value of 3.27 µM [120].

**FIGURE 16:**
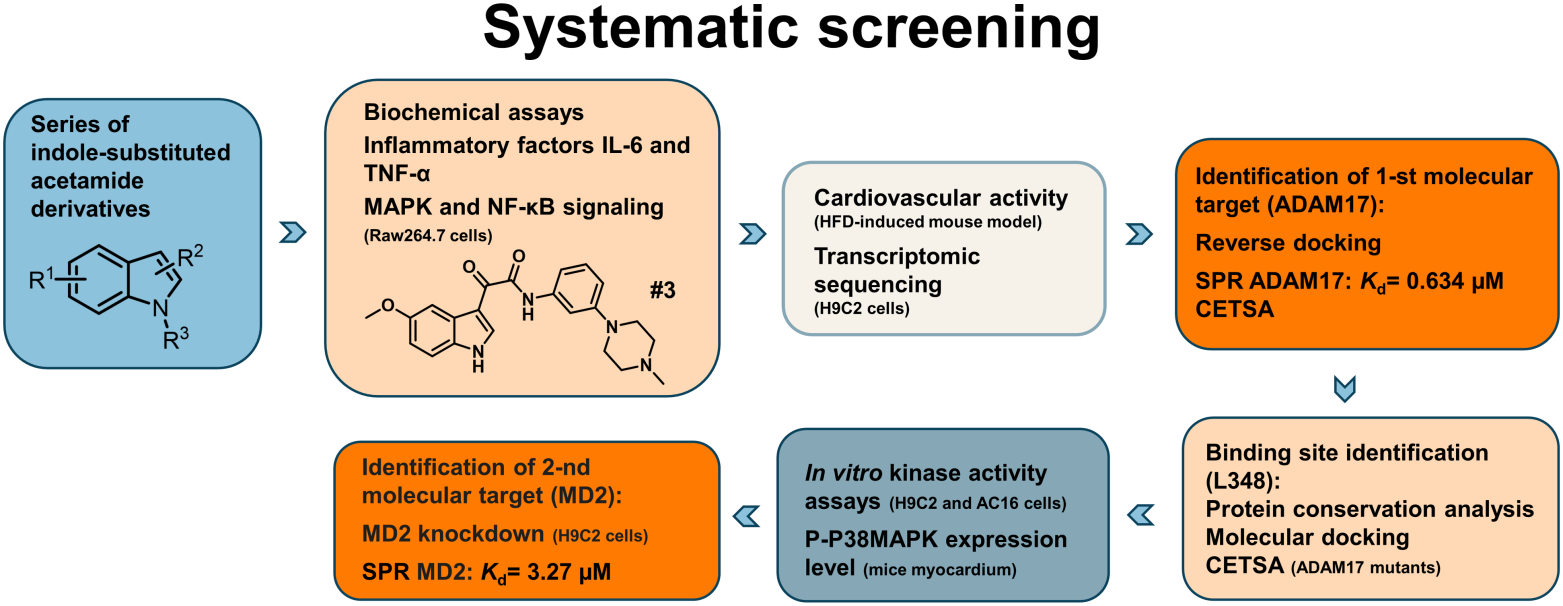
Systematic screening approach for developing MD2/ADAM17 dual‐target inhibitors [120]. Compound **#3** was pre‐selected during the screening of a series of acetamide derivatives with indole substituents in biochemical assays on inflammatory factors. Molecular targets were identified through subsequent studies of cardiovascular activity, molecular docking, analysis of protein expression levels, and kinase activity.

Overall, although screening remains the primary method in the early stages of drug discovery, it requires significant resources, including high consumption of chemical compounds, infrastructure, and subsequent validation, which slows the “hit‐to‐lead” transition. Furthermore, systematic screening rarely considers factors such as tissue‐specific target expression, metabolic stability, or pharmacokinetic limitations, leading to high attrition rates when promising hits fail to translate into viable clinical candidates.

## 7 Summary and Outlook

The development of MTDs offers a strategy for treating complex diseases, including cardiovascular disorders. Progress in this area is supported by integrating medicinal chemistry with computational methodologies, including AI, which is reshaping target identification, molecular design, and optimization of pharmacological properties.

Unlike conventional combination therapy with multiple single‐target drugs, multitarget therapeutics provide an integrated approach to cardiovascular disease management by minimizing adverse drug–drug interactions. This strategy is particularly advantageous for multifactorial conditions, such as AF, heart failure, and myocardial ischemia–reperfusion injury [6, 123]. MTDs can simultaneously act on different biological pathways involved in heart and skeletal muscle diseases, enhancing treatment efficacy over single‐target drugs. They may improve patient outcomes by potentially providing additive or synergistic cardioprotective effects, especially in patients with comorbidities such as diabetes, hypertension, and hyperlipidemia [123]. Multitarget therapies have the potential to more effectively address the complex pathophysiological mechanisms underlying CVDs, including metabolic disturbances, inflammatory activation, cellular injury, and maladaptive remodeling processes [123]. Compared with drug combinations, MTDs have more predictable pharmacokinetics and minimize drug–drug interactions [6]. Consequently, MTDs may complement or even replace conventional combination therapies by integrating the beneficial effects of multiple mechanism‐based interventions within a single molecular entity. Because many cardiac patients have diabetes, obesity, chronic kidney disease, or age‐related heart changes, MTDs affecting several disease‐related pathways may offer more robust protection than single‐target drugs [123, 124, 125].

When comparing various strategies for developing MTDs, it is important to note that the choice of approach depends on the disease context, selected target combination, availability of published data, and drug discovery stage. For example, rational design and systematic screening may serve as suitable starting points, whereas molecular hybridization is generally more useful during lead optimization than in the early stages of hit identification (Table 1).

**TABLE 1:** Comparison of methods.

Method	Most suitable for	Typical stage
Molecular hybridization	Combining proven fragments into potent multitarget leads	Lead optimization
Polypharmacology/ network pharmacology	Identification of new off‐target sites for currently available drugs	Target selection and drug design
SOSA	Turning weak off‐targets into new indications	Lead optimization
Peptide therapeutics	Complicated target sites requiring high specificity	Preclinical to clinical
Drug repurposing	Rapid trial progression with low risk	Late‐stage discovery/clinical
Rational design	High quality targets with known structures	Hit‐to‐lead optimization
Systematic screening	Unbiased hit discovery in large libraries	Early discovery

Several antiarrhythmic agents with multitarget mechanisms of action are currently available for the treatment of AF; however, their clinical use remains limited by suboptimal efficacy, adverse effects, and safety concerns. Amiodarone is an aryl‐phenylketone that blocks multiple ion channels and adrenergic receptors, including Na^+^/K^+^/L‐type Ca^2+^channels and beta‐adrenergic receptors. This broad pharmacological profile makes it effective for rhythm control; however, its usage is limited by the risk of potential severe toxicities and significant drug–drug interactions (Figures 1 and 17) [10, 14, 131]. Dronedarone, a derivative of amiodarone, is associated with better safety profile but reduced efficacy (Figure 17) [126, 132]. Budiodarone, another amiodarone analog, is associated with fewer adverse effects due to its more rapid metabolism (Figure 17) [126, 130]. It has shown promising results in Phase II clinical trials, and is currently in Phase III [129, 131]. Presentation data and trial design for the concurrent open‐label HARBOR‐AF study involving 1000 subjects with proximal AF were published in November 2025; however, interim results are still pending [131].

**FIGURE 17:**
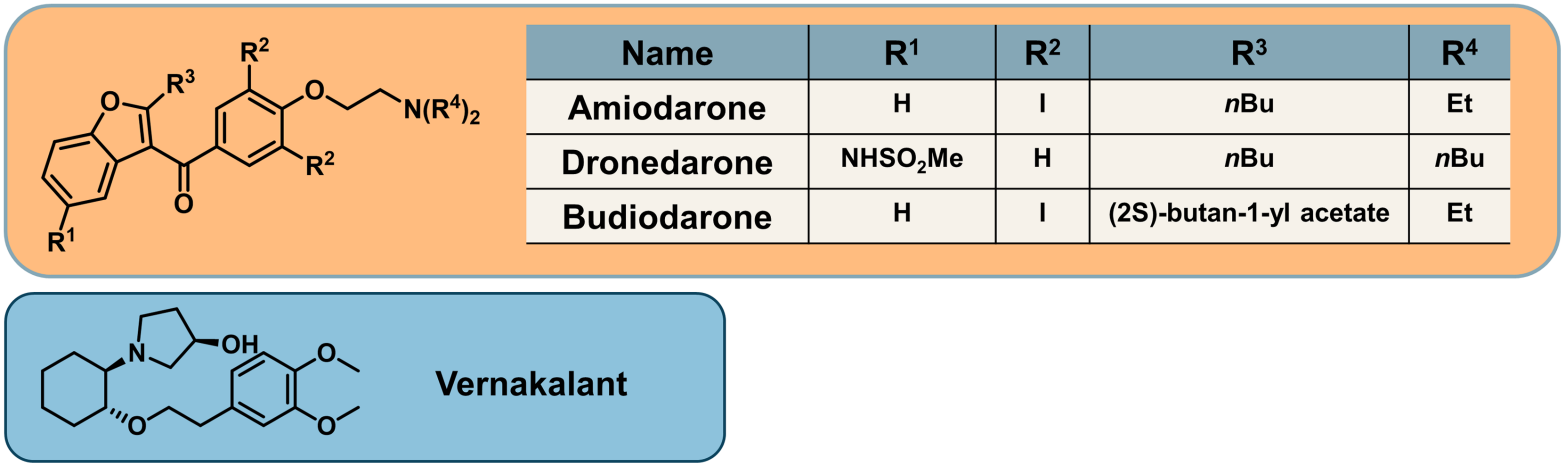
The structures of amiodarone and its analogs, as well as vernakalant [14, 126, 130].

Vernakalant (approved in Europe as Brinavess, Figure 17) is a pyrrolidine compound that is structurally unrelated to amiodarone [126]. Its antiarrhythmic activity results from the modulation of several atrial ion currents, including I_Kur_, I_K,ACh_, I_to_, I_Kr_, and frequency‐dependent sodium currents, leading to effects on both atrial conduction and refractoriness [128]. A distinctive feature of vernakalant is its rate‐dependent interaction with sodium channels, whereby the binding affinity increases at higher heart rates, enabling rapid restoration of sinus rhythm [133, 134, 135]. Although vernakalant is generally considered to have a more favorable safety profile than amiodarone, it may still be associated with clinically significant adverse effects, such as hypotension, bradycardia, and, rarely, complete heart block [133]. Owing to concerns regarding cardiovascular safety, the Food and Drug Administration (FDA) has denied approval of this drug in the United States [136]. Table 2 provides an overview of the compounds discussed, their evaluation stages, and the main conclusions.

**TABLE 2:** Overview of the discussed compounds, their evaluation stages, and the key findings.

Compound^a^	Targets	Evaluation stage	Key findings	References
**PTPUP**	COX‐2, sEH	In vitro and in vivo (murine models)	Potent dual inhibitory activity, anti‐inflammatory activity	[34]
**12, 13**	RyR2, SERCA2, Na_V_1,5	In vitro	Triple activity: RyR2‐ stabilizing, SERCA2 activating and Na_V_1.5 blocking	[39]
**Codrug 3b**	NEP, AT1	In vitro and in vivo (murine model)	AT1 antagonist and NEP inhibitor. Systolic and diastolic blood pressure reduction, antihypertensive effects	[53]
**2b**	Na^+^‐channels, ROS scavenger	In vitro and ex vivo (rodent model)	Superior antiarrhythmic profile versus mexiletine; antioxidant activity	[56]
**4i**	Nrf2, Ca_V_1.x, Cat S	In vitro	Modest triple activity: activator of Nrf2 antioxidant pathways, Ca_V_1.x blocker, Cat S inhibitor	[66]
**C165, C115, T3, T4, TD104, TD106**	ACE, NEP	In silico, virtual screening	Predicted dual‐target inhibitory activity, no experimental validation reported	[74]
**7, 8, 9**	PPARδ, sEH	In silico and in vitro	Dual‐target activity: PPARδ agonists, sEH inhibitors	[76]
**6f**	K_V_1.5, Na_V_1.5, TASK‐1	In silico and in vitro (human cardiomyocytes)	Multitarget inhibitory activity	[81]
**DHP‐57**	Ca_V_1.2, K_Ca_3.1	In vitro	Dual inhibitory activity	[84]
**MT‐1207**	α_1A_‐, α_1B_‐_,_ α_1D_‐AR, 5‐HT2A	In vitro and in vivo (murine models), clinical (Phase II)	Potent multitarget inhibitory activity, antihypertensive drug candidate	[89]
**S100A1ct**	RyR2, SERCA2a	In vitro and in vivo (murine and porcine models)	Dual‐target activity: SERCA2a activation, RyR2 stabilizer	[95]
**1**	Ang II, Ca_V_1.x	In silico, ex vivo, in vivo (murine model)	Dual activity: Ang II antagonist, Ca_V_1.x blocker. Vasorelaxant and antihypertensive effect	[108]
**GD4, GD10**	Ca_V_1.2, Ca_V_1.3	In vitro	L‐ and T‐type calcium channel blockers	[113]
**GM1869**	RyR2, SERCA2a	In vitro, in vivo (currently running, murine model)	Dual‐target activity: SERCA2a activation, RyR2 stabilizer	[116, 117]
**#3**	MD2, ADAM17	In vitro, in vivo (murine model)	Dual inhibitory activity, improved cardiac function anti‐inflammatory profile	[120]
**Amiodarone**	Na^+^, K^+^, L‐type Ca^2+^‐channels, β‐AR	Approved drug	Sinus rhythm restoration, risk of potential severe toxicities, drug–drug interactions	[132, 10, 14]
**Dronedarone**	Na^+^, K^+^, L‐type Ca^2+^‐channels, β‐AR	Postmarketing surveillance Phase IV	Sinus rhythm restoration; reduced efficacy versus amiodarone	[126, 127]
**Budiodarone**	Na^+^, K^+^, L‐type Ca^2+^‐channels, β‐AR	Clinical (Phase III)	Heart rhythm stabilization; favorable safety versus amiodarone	[126, 129, 131]
**Vernakalant**	I_Kur_, I_K,ACh_, I_to_, I_Kr_, I_Na_, late I_Na_	Approved in Europe	Rapid sinus rhythm restoration; favorable safety versus amiodarone	[128]

a All compound names are retained as originally published.

Despite the compelling preclinical rationale, several translational hurdles remain before multitarget cardiovascular therapeutics can achieve widespread clinical adoption. Multiple targets often exhibit divergent tissue distributions, half‐lives, and activation thresholds. Therefore, it is challenging to determine a dose that would simultaneously affect all target systems without causing any toxicity. In addition, regulatory procedures and frameworks for conducting clinical trials remain largely optimized for drugs that target a single mechanism of action. Adapting them to account for multitarget mechanisms of action will require the use of new biomarker strategies, adaptive trial designs, and thorough assessment of the benefit–risk profile. Due to the diverse nature of CVDs, it is essential to precisely categorize patients and employ supplementary diagnostic tests to determine which population groups will gain the most clinical advantage. Finally, robust long‐term outcome data from large‐scale phase III trials are still limited, underscoring the need for dedicated clinical programs that evaluate not only efficacy but also safety, tolerability, and real‐world effectiveness. Addressing these translational challenges is essential for realizing the full clinical potential of multitarget therapeutics in cardiology.

## Funding

This work was supported by German Centre for Cardiovascular Research (DZHK).

## Conflicts of Interest

The authors declare no conflicts of interest.

## Data Availability

The data are already available in the cited references.
